# Platelet RNA enables accurate detection of ovarian cancer: an intercontinental, biomarker identification study

**DOI:** 10.1093/procel/pwac056

**Published:** 2022-11-10

**Authors:** Yue Gao, Chun-Jie Liu, Hua-Yi Li, Xiao-Ming Xiong, Gui-Ling Li, Sjors G J G In ‘t Veld, Guang-Yao Cai, Gui-Yan Xie, Shao-Qing Zeng, Yuan Wu, Jian-Hua Chi, Jia-Hao Liu, Qiong Zhang, Xiao-Fei Jiao, Lin-Li Shi, Wan-Rong Lu, Wei-Guo Lv, Xing-Sheng Yang, Jurgen M J Piek, Cornelis D de Kroon, C A R Lok, Anna Supernat, Sylwia Łapińska-Szumczyk, Anna Łojkowska, Anna J Żaczek, Jacek Jassem, Bakhos A Tannous, Nik Sol, Edward Post, Myron G Best, Bei-Hua Kong, Xing Xie, Ding Ma, Thomas Wurdinger, An-Yuan Guo, Qing-Lei Gao

**Affiliations:** Department of Gynecological Oncology, Tongji Hospital, Tongji Medical College, Huazhong University of Science and Technology, Wuhan 430030, China; National Clinical Research Center for Obstetrics and Gynecology, Cancer Biology Research Center (Key Laboratory of the Ministry of Education), Tongji Hospital, Tongji Medical College, Huazhong University of Science and Technology, Wuhan 430030, China; Center for Artificial Intelligence Biology, Hubei Bioinformatics & Molecular Imaging Key Laboratory, Key Laboratory of Molecular Biophysics of the Ministry of Education, College of Life Science and Technology, Huazhong University of Science and Technology, Wuhan 430074, China; Department of Gynecological Oncology, Tongji Hospital, Tongji Medical College, Huazhong University of Science and Technology, Wuhan 430030, China; National Clinical Research Center for Obstetrics and Gynecology, Cancer Biology Research Center (Key Laboratory of the Ministry of Education), Tongji Hospital, Tongji Medical College, Huazhong University of Science and Technology, Wuhan 430030, China; Department of Gynecological Oncology, Tongji Hospital, Tongji Medical College, Huazhong University of Science and Technology, Wuhan 430030, China; National Clinical Research Center for Obstetrics and Gynecology, Cancer Biology Research Center (Key Laboratory of the Ministry of Education), Tongji Hospital, Tongji Medical College, Huazhong University of Science and Technology, Wuhan 430030, China; Department of Obstetrics and Gynecology, The First Affiliated Hospital of Nanchang University, Nanchang 330006, China; Cancer Center, Union Hospital, Tongji Medical College, Huazhong University of Science and Technology, Wuhan 430022, China; Department of Neurosurgery, Amsterdam UMC, VU University Medical Center, Cancer Center Amsterdam, De Boelelaan 1117, 1081 HV Amsterdam, The Netherlands; Brain Tumor Center Amsterdam, Amsterdam UMC, VU University Medical Center, Cancer Center Amsterdam, De Boelelaan 1117, 1081 HV Amsterdam, The Netherlands; Department of Gynecological Oncology, Tongji Hospital, Tongji Medical College, Huazhong University of Science and Technology, Wuhan 430030, China; National Clinical Research Center for Obstetrics and Gynecology, Cancer Biology Research Center (Key Laboratory of the Ministry of Education), Tongji Hospital, Tongji Medical College, Huazhong University of Science and Technology, Wuhan 430030, China; Center for Artificial Intelligence Biology, Hubei Bioinformatics & Molecular Imaging Key Laboratory, Key Laboratory of Molecular Biophysics of the Ministry of Education, College of Life Science and Technology, Huazhong University of Science and Technology, Wuhan 430074, China; Department of Gynecological Oncology, Tongji Hospital, Tongji Medical College, Huazhong University of Science and Technology, Wuhan 430030, China; National Clinical Research Center for Obstetrics and Gynecology, Cancer Biology Research Center (Key Laboratory of the Ministry of Education), Tongji Hospital, Tongji Medical College, Huazhong University of Science and Technology, Wuhan 430030, China; Department of Gynecological Oncology, Tongji Hospital, Tongji Medical College, Huazhong University of Science and Technology, Wuhan 430030, China; National Clinical Research Center for Obstetrics and Gynecology, Cancer Biology Research Center (Key Laboratory of the Ministry of Education), Tongji Hospital, Tongji Medical College, Huazhong University of Science and Technology, Wuhan 430030, China; Department of Gynecological Oncology, Tongji Hospital, Tongji Medical College, Huazhong University of Science and Technology, Wuhan 430030, China; National Clinical Research Center for Obstetrics and Gynecology, Cancer Biology Research Center (Key Laboratory of the Ministry of Education), Tongji Hospital, Tongji Medical College, Huazhong University of Science and Technology, Wuhan 430030, China; Department of Gynecological Oncology, Tongji Hospital, Tongji Medical College, Huazhong University of Science and Technology, Wuhan 430030, China; National Clinical Research Center for Obstetrics and Gynecology, Cancer Biology Research Center (Key Laboratory of the Ministry of Education), Tongji Hospital, Tongji Medical College, Huazhong University of Science and Technology, Wuhan 430030, China; Center for Artificial Intelligence Biology, Hubei Bioinformatics & Molecular Imaging Key Laboratory, Key Laboratory of Molecular Biophysics of the Ministry of Education, College of Life Science and Technology, Huazhong University of Science and Technology, Wuhan 430074, China; Department of Gynecological Oncology, Tongji Hospital, Tongji Medical College, Huazhong University of Science and Technology, Wuhan 430030, China; National Clinical Research Center for Obstetrics and Gynecology, Cancer Biology Research Center (Key Laboratory of the Ministry of Education), Tongji Hospital, Tongji Medical College, Huazhong University of Science and Technology, Wuhan 430030, China; Cancer Center, Union Hospital, Tongji Medical College, Huazhong University of Science and Technology, Wuhan 430022, China; Department of Gynecological Oncology, Tongji Hospital, Tongji Medical College, Huazhong University of Science and Technology, Wuhan 430030, China; National Clinical Research Center for Obstetrics and Gynecology, Cancer Biology Research Center (Key Laboratory of the Ministry of Education), Tongji Hospital, Tongji Medical College, Huazhong University of Science and Technology, Wuhan 430030, China; Department of Gynecological Oncology, Women’s Hospital, School of Medicine, Zhejiang University, Hangzhou 310011, China; Gynecological Oncology Key Laboratory, Qilu Hospital, Shandong University, Jinan 250100, China; Department of Obstetrics and Gynecology, Catharina Hospital, Michelangelolaan 2, 5623EJ Eindhoven, Eindhoven, The Netherlands; Department of Obstetrics and Gynecology, Leiden University Medical Center, Albinusdreef 2, 2300 RC, Leiden, The Netherlands; Department of Gynecological Oncology, Center of Gynecologic Oncology Amsterdam, Antoni van Leeuwenhoek, Plesmanlaan 121, 1066 CX Amsterdam, The Netherlands; Laboratory of Translational Oncology, Intercollegiate Faculty of Biotechnology, University of Gdańsk and Medical University of Gdańsk, Gdańsk, Poland; Department of Gynecology, Gynecological Oncology and Gynecological Endocrinology, Medical University of Gdańsk, Gdańsk, Poland; Department of Gynecology, Gynecological Oncology and Gynecological Endocrinology, Medical University of Gdańsk, Gdańsk, Poland; Laboratory of Translational Oncology, Intercollegiate Faculty of Biotechnology, University of Gdańsk and Medical University of Gdańsk, Gdańsk, Poland; Department of Oncology and Radiotherapy, Medical University of Gdańsk, Gdańsk, Poland; Department of Neurology, Massachusetts General Hospital and Neuroscience Program, Harvard Medical School, 149 13th Street, Charlestown, MA 02129, USA; Department of Neurosurgery, Amsterdam UMC, VU University Medical Center, Cancer Center Amsterdam, De Boelelaan 1117, 1081 HV Amsterdam, The Netherlands; Department of Neurology, Amsterdam UMC, VU University Medical Center, Cancer Center Amsterdam, De Boelelaan 1117, 1081 HV Amsterdam, The Netherlands; Department of Neurosurgery, Amsterdam UMC, VU University Medical Center, Cancer Center Amsterdam, De Boelelaan 1117, 1081 HV Amsterdam, The Netherlands; Department of Neurosurgery, Amsterdam UMC, VU University Medical Center, Cancer Center Amsterdam, De Boelelaan 1117, 1081 HV Amsterdam, The Netherlands; Gynecological Oncology Key Laboratory, Qilu Hospital, Shandong University, Jinan 250100, China; Department of Gynecological Oncology, Women’s Hospital, School of Medicine, Zhejiang University, Hangzhou 310011, China; Department of Gynecological Oncology, Tongji Hospital, Tongji Medical College, Huazhong University of Science and Technology, Wuhan 430030, China; National Clinical Research Center for Obstetrics and Gynecology, Cancer Biology Research Center (Key Laboratory of the Ministry of Education), Tongji Hospital, Tongji Medical College, Huazhong University of Science and Technology, Wuhan 430030, China; Department of Neurosurgery, Amsterdam UMC, VU University Medical Center, Cancer Center Amsterdam, De Boelelaan 1117, 1081 HV Amsterdam, The Netherlands; Center for Artificial Intelligence Biology, Hubei Bioinformatics & Molecular Imaging Key Laboratory, Key Laboratory of Molecular Biophysics of the Ministry of Education, College of Life Science and Technology, Huazhong University of Science and Technology, Wuhan 430074, China; Department of Gynecological Oncology, Tongji Hospital, Tongji Medical College, Huazhong University of Science and Technology, Wuhan 430030, China; National Clinical Research Center for Obstetrics and Gynecology, Cancer Biology Research Center (Key Laboratory of the Ministry of Education), Tongji Hospital, Tongji Medical College, Huazhong University of Science and Technology, Wuhan 430030, China

**Keywords:** tumor-educated platelets, ovarian cancer, liquid biopsy, preoperative diagnosis

## Abstract

Platelets are reprogrammed by cancer via a process called education, which favors cancer development. The transcriptional profile of tumor-educated platelets (TEPs) is skewed and therefore practicable for cancer detection. This intercontinental, hospital-based, diagnostic study included 761 treatment-naïve inpatients with histologically confirmed adnexal masses and 167 healthy controls from nine medical centers (China, *n* = 3; Netherlands, *n* = 5; Poland, *n* = 1) between September 2016 and May 2019. The main outcomes were the performance of TEPs and their combination with CA125 in two Chinese (VC1 and VC2) and the European (VC3) validation cohorts collectively and independently. Exploratory outcome was the value of TEPs in public pan-cancer platelet transcriptome datasets. The AUCs for TEPs in the combined validation cohort, VC1, VC2, and VC3 were 0.918 (95% CI 0.889–0.948), 0.923 (0.855–0.990), 0.918 (0.872–0.963), and 0.887 (0.813–0.960), respectively. Combination of TEPs and CA125 demonstrated an AUC of 0.922 (0.889–0.955) in the combined validation cohort; 0.955 (0.912–0.997) in VC1; 0.939 (0.901–0.977) in VC2; 0.917 (0.824–1.000) in VC3. For subgroup analysis, TEPs exhibited an AUC of 0.858, 0.859, and 0.920 to detect early-stage, borderline, non-epithelial diseases and 0.899 to discriminate ovarian cancer from endometriosis. TEPs had robustness, compatibility, and universality for preoperative diagnosis of ovarian cancer since it withstood validations in populations of different ethnicities, heterogeneous histological subtypes, and early-stage ovarian cancer. However, these observations warrant prospective validations in a larger population before clinical utilities.

## Introduction

Beyond the salient functions in hemostasis and thrombosis, platelets play significant roles in fostering cancer progression including tumor growth, angiogenesis, immune evasion, and most notably, metastasis (Haemmerle et al., 2018). The interface between platelets and cancer cells is bidirectional (López, 2021). Products of circulating tumor cells can be sequestered within platelets due to their capabilities of endocytosis as sentinels of circulation (Xu et al., 2018). Via a series of events called education, cancer cells and their creations inflict considerable phenotypic modulations on platelets in terms of biological behaviors, numbers, and contents such as RNAs, which could be leveraged to seek and characterize cancer (In ‘t Veld and Wurdinger, 2019; Roweth and Battinelli, 2021). Thrombocytosis is associated with increased risk and shortened survival of cancer, especially in ovarian cancer (Stone et al., 2012; Giannakeas and Narod, 2021). RNA sequencing of TEPs has become the latest component of liquid biopsy for cancer detection since TEPs are transcriptionally reprogrammed by tumor cells through three main mechanisms including inducing RNA splicing, stimulating translation and subsequent RNA decay, and sequestration and release of RNA in circulation (D’Ambrosi et al., 2021). Altered counts, functions, and highly dynamic RNA repertoire of platelets provide a substantial basis for utilizing their RNA profiles to detect ovarian cancer. TEPs potentially provides a promising source for detection of cancer.

The crosstalk between platelets and cancer underscores both the protumor effects and cancer-induced alternations within platelets. A resounding example to illustrate the interplay is paraneoplastic thrombocytosis in ovarian cancer. Cancer cells autonomously secret interleukin-6 to increase hepatic thrombopoietin synthesis and induce paraneoplastic thrombocytosis, which associates with advanced disease (Stone et al., 2012). Mechanistically, platelets can increase proliferation (Cho et al., 2012), promote tissue evasion and metastasis (Haemmerle et al., 2017), and bolster angiogenesis in ovarian cancer (Klement et al., 2009). Major morphological changes in the organelles of platelets isolated from patients with ovarian cancer indicate rewired functions of platelets associated with this malignancy (Wang et al., 2015). Currently, ovarian cancer management is riddled with challenges to reduce its stubbornly high mortality, where applications of TEPs might give rise to improvements. Ovarian cancer is the fifth leading cause of cancer-related deaths in women and the most lethal gynecological cancer that often evades early detection and defies treatment (Lheureux et al., 2019b; Siegel et al., 2021). Around 70% of patients with ovarian cancer are initially diagnosed at an advanced stage, which have a 5-year relative survival rate of 30%, in contrast with 93% for a localized disease (Siegel et al., 2021). Since ovarian cancer screenings are not recommended in average-risk, asymptomatic populations (Henderson et al., 2018; Menon et al., 2021), enabling early detection of ovarian cancer is an important avenue to better prognosis (Vaughan et al., 2011). The scarcity of accurate methods for detecting early-stage curable disease thwarts clinicians and researchers, which calls for novel tools. CA125 is the most clinically used biomarker for ovarian cancer, but its sensitivity and specificity for detection of ovarian cancer remain unmet clinically. Patients with borderline ovarian tumors and non-epithelial ovarian malignancies often harbored normal CA125, while many benign adnexal lesions such as endometriosis are accompanied by increased CA125.

Liquid biopsies based on circulating tumor DNA (ctDNA), circulating tumor cells (CTCs), exosomes, or tumor-educated platelet (TEPs) distinguish from direct tumor biopsies for their minimal invasiveness, dynamic sampling, and comprehensive molecular profiling. Plasma ctDNA, CTCs, and exosomal miRNAs has achieved promising diagnostic accuracy over cancer antigen 125 (CA125) in small groups of patients with ovarian cancer, among which ctDNA demonstrates the highest accuracy. However, whether TEPs could enable accurate detection of ovarian cancer remains unknown.

In this study, we built a classifier based on platelet RNA profiles that enabled early and accurate detection of ovarian cancer and validated it in two Chinese and one European external validation cohorts. By comparing the classifier with carbohydrate antigen 125 (CA125), a commonly used biomarker for ovarian cancer, we found that the classifier displayed improved diagnostic capabilities for early-stage disease and borderline ovarian tumors in three external validation cohorts, and for non-epithelial ovarian malignancies and discriminating ovarian cancer from endometriosis in two Chinese external validation cohorts. Moreover, a combination of CA125 with the classifier further augmented the diagnostic performance. This study confirms the potential of platelet RNA profiling and provides an applicable classifier for blood-based cancer detection.

## Results

### Diagnostic potential of platelet RNA in ovarian cancer

**Figure 1.**
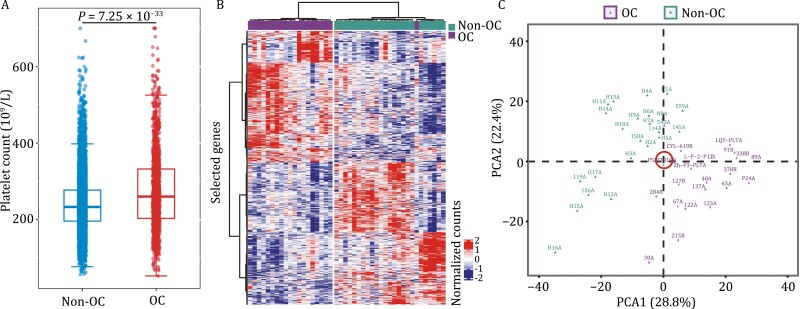
**Differences in platelets between OC and non-OC patients.** (A) The real-world study of the association between platelet counts and ovarian cancer. These individuals were composed of 1,301 patients with ovarian cancer and 8,667 individuals without cancer Boxplots represent median value, with lower and upper hinges corresponding to the 25th and 75th percentiles, and lower and upper whiskers extending from the hinge to the smallest and largest value at most 1.5× interquartile range of the hinge, respectively. Two-sided student’s *t*-test. (B) Heatmap of unsupervised clustering and (C) platelet mRNA profiles of patients with ovarian cancer and non-OC individuals in discovery cohort. We found that mRNA sequencing can well discriminate patients with OC from non-OC individuals. OC, ovarian cancer.

Paraneoplastic thrombocytosis in ovarian cancer was confirmed by our real-world evidence. We extracted platelet counts of 9,968 individuals from China Real World Gynecological Oncology Platform between September 2016 to May 2019 [Qilu Hospital of Shandong University, *n* = 1,067 (ovarian cancer, *n* = 48); Tongji Hospital, *n* = 3,279 (ovarian cancer, *n* = 790); Women’s Hospital of Zhejiang University, *n* = 5,622 (ovarian cancer, *n* = 463)]. Platelet counts of 1,301 patients with ovarian cancer (OC) and 8,667 non-OC individuals enrolled from three hospitals were analyzed. As a result, we observed a significantly higher platelet count for patients with OC than non-OC individuals (Fig. 1A). To prove the association between platelet transcriptome and ovarian cancer, we performed RNA-sequencing of TEPs in discovery cohort including 44 individuals (ovarian cancer, *n* = 20; BAM, *n* = 7; healthy women, *n* = 17). Unsupervised hierarchical clustering heatmap (Fig. 1B) and PCA (principal component analysis) (Fig. 1C) analysis showed that mRNA sequencing of TEPs enabled accurate discriminations between patients with ovarian cancer and individuals without cancer.

### Participants and their baseline characteristics

**Figure 2.**
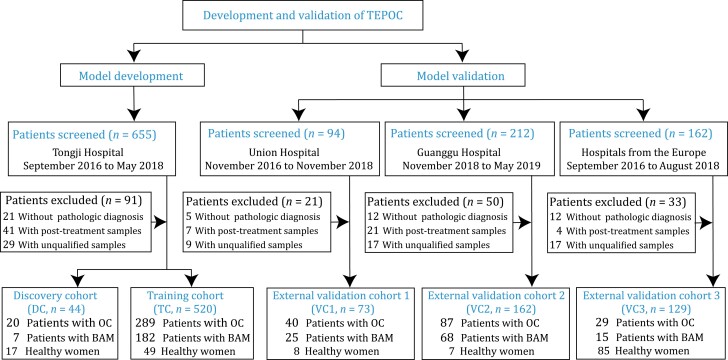
**Study design and patient enrollment.** Unqualified samples were those with low quality (RNA integrity number < 7) or quantity (<10 pg) of total RNA. OC, ovarian cancer. BAM, benign adnexal mass. TEPOC, tumor-educated platelet-derived gene panel of ovarian cancer. TC, training cohort. DC, discovery cohort. VC1, validation cohort 1. VC2, validation cohort 2. VC3, validation cohort 3.

The study design was presented in Fig. 2. The proportion of ovarian cancer patients in five cohorts was 55.6%, 45.5%, 54.8%, 53.7%, and 22.5%, respectively. The median age of the participants was 47.0 years (interquartile range [IQR]: 40.8–54) in the training cohort, 44.5 years (IQR: 40.0–54.0) in the discovery cohort, 50.0 years (IQR: 42.0–54.0) in the validation cohort 1, 48.0 years (IQR: 39.0–55.0) in the validation cohort 2, and 55.0 years (IQR: 42.0–63.0) in the validation cohort 3. Collectively, the proportion of ovarian cancer patients in the European cohort was lower than that in the Chinese cohorts, and the median age of the participants in the European cohort was higher than that in the Chinese cohorts. Other baseline characteristics of participants are provided in Table 1.

**Table 1.** Patient characteristics at baseline.

		Training cohort	Discovery cohort	Validation cohort 1	Validation cohort 2	Validation cohort 3
Group	OC, *n*	289 (55.6%)	20 (45.5%)	40 (54.8%)	87 (53.7%)	29 (22.5%)
	Non-OC	231 (44.4%)	24 (54.5%)	33 (45.2%)	75 (46.3%)	100 (77.5%)
	BAM, *n*	182	7	25	68	14
	Healthy women, *n*	49	17	8	7	86
	Total, *n*	520	44	73	162	129
Age, median, year		47.0 (40.8–54.0)	44.5 (40.0–54.0)	50.0 (42.0–54.0)	48.0 (39.0–55.0)	55.0 (42.0–63.0)
*OC histology*						
Epithelial	Serous, *n*	178	12	24	40	18
	Mucinous, *n*	11	0	1	0	0
	Endometrioid, *n*	23	1	3	1	1
	Clear cell, *n*	11	1	0	1	1
	Borderline, *n*	52	4	10	24	9
Non-epithelial	*N*	14	2	2	21	0
BAM histology						
	Endometriosis, *n*	57	6	9	28	0
	Others*, *n*	125	1	16	40	14
	Total, *n*	182	7	25	68	14
OC FIGO stage						
	Early stage (I&IIA), *n*	49	3	7	23	11
	Late stage (IIB-IV), *n*	240	17	33	64	18
	Total, *n*	289	20	40	87	29
OC grade	Well	36	3	6	12	4
	Moderate	48	2	4	12	5
	Poor	205	15	30	63	20
CA125 level						
	<35 mU/L, *n*	196	11	28	56	6
	≥35 mU/L, *n*	324	33	45	106	29
	Missing, *n*	0	0	0	0	94
	Total, *n*	520	44	73	162	129

Abbreviations: OC, ovarian cancer; BAM, benign adnexal mass; CA125, cancer antigen 125; FIGO, International Federation of Gynecology and Obstetrics. *The details of BAMs besides endometriosis are presented in Table S1.

### Model development

Samples in training and validation cohorts were subsequently sequenced at the mRNA level for analysis according to Fig. 1B and 1C. Sequencing data in the training cohort showed that the number of high-confidence genes (>30 reads) was significantly greater in ovarian cancer samples (median value, 8,054) than in control samples (median value, 7,605) (*P* = 0.018; Fig. S3A). Five genes with the highest expression were MT-RNR2, MT-RNR1, TMSB4X, B2M, and MTND1 (Fig. S3B). Data processing was performed to eliminate batch effects and to identify and adjust for confounding variables such as age and total read count (Fig. S4A–D). Model development and optimization workflow is pictorially shown in Fig. S5, which generated a classifier of 102 platelet RNAs named TEP-derived gene panel of ovarian cancer (TEPOC). The contributing gene list, location, and description are presented in Table S2. To gain a better insight into the biological relevance, these feature genes were enriched through MSigDB cancer hallmark and C6 oncogenic signature gene set collections by clusterProfiler as illustrated in Fig. S6. Ovarian cancer is known to predispose to pelvic metastasis, particularly through the adipose tissue-rich omentum (Motohara et al., 2019). Among the 102 feature genes, eight genes (MYLK, GADD45A, ACADM, UQCR11, TST, PTCD3, SOD1, CD151) were significantly enriched in the cancer hallmark of adipogenesis. Five feature genes were the up-regulation of placental growth factor (PGF), and PGF was reported to associate with unfavorable prognosis of ovarian cancer (Meng et al., 2018). The panel gene GADD45A was significantly related to adipogenesis, apoptosis, E2F1, and HOXA9 at the same time. Studies indicated GADD45A was associated with ovarian cancer susceptibility and prognosis (Yuan et al., 2015). We also compared the 1625 differentially expressed genes in the lung cancer diagnosis with our 102 panel genes and found there were 29 overlapped genes. To our great surprise, the top cancer hallmark and oncogenic signature genes enriched in TEPOC (Fig. S6) and the lung cancer panel (Fig. S7) were consistent.

### Model performance and its combination with CA125 in validation cohorts

**Figure 3.**
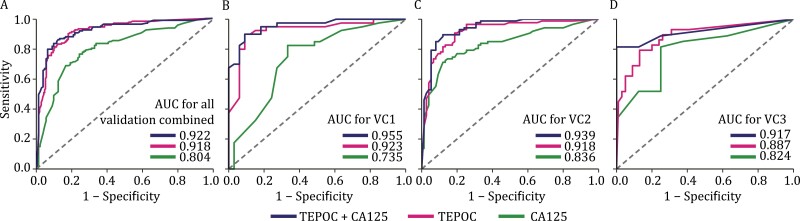
**Performance of TEPOC and its combination with CA125 in validation cohorts and ovarian cancer subgroups.** The performance of CA125 (green line), TEPOC (red line), and TEPOC+CA125 (blue line) to detect ovarian cancer were estimated by plotting receiver operating characteristic curves in (A) three validation cohorts combined, (B) validation cohort 1, (C) validation cohort 2, (D) validation cohort 3. Abbreviations: AUC, area under the curve; TEPOC, tumor-educated platelet-derived gene panel of ovarian cancer; CA125, cancer antigen 125; TEPOC+CA125, a combined diagnosis of TEPOC and CA125; BAM, benign adnexal mass.

We assessed TEPOC in three validation cohorts collectively and independently (Fig. 3; Table 2). Its AUC to detect ovarian cancer in the combined validation cohorts was 0.918 (95% CI 0.889–0.948). The combinatorial diagnosis using TEPOC and CA125 resulted in an AUC of 0.922 (95% CI 0.889–0.955). Both TEPOC (*P* < 0.001) and the combination (*P* < 0.001) exhibited significantly greater diagnostic AUCs than CA125 [0.804 (95% CI 0.750–0.857)] (Fig. 3A).

**Table 2.** Performance of TEPOC and CA125 to detect ovarian cancer in validation cohorts.

	AUC (95% CI)	ACC (95% CI), %	SN (95% CI), %	SP (95% CI), %	PPV (95% CI), %	NPV (95% CI), %	Kappa	F1	AUC *P*-value
*All validation*									
TEPOC	0.918 (0.889–0.948)	83.8 (79.6–87.4)	85.3 (78.7–90.4)	82.7 (76.9–87.6)	78.7 (71.7–84.6)	88.2 (82.8–92.4)	0.672	0.818	<0.001
CA125	0.804 (0.750–0.857)	75.9 (70.4–80.9)	78.6 (71.2–84.8)	72.4 (63.3–80.3)	79.1 (71.8–85.2)	71.8 (62.7–79.7)	0.509	0.788	—
TEPOC+CA125	0.922 (0.889–0.955)	85.9 (81.2–89.8)	86.4 (79.9–91.4)	85.3 (77.6–91.2)	88.7 (82.5–93.3)	82.5 (74.5–88.8)	0.714	0.875	<0.001
*VC1*									
TEPOC	0.923 (0.855–0.990)	84.9 (74.6–92.2)	95.0 (83.1–99.4)	72.7 (54.5–86.7)	80.9 (66.7–90.9)	92.3 (74.9–99.1)	0.690	0.874	0.013
CA125	0.735 (0.615–0.855)	75.3 (63.9–84.7)	82.5 (67.2–92.7)	66.7 (48.2–82.0)	75.0 (59.7–86.8)	75.9 (56.5–89.7)	0.497	0.786	—
TEPOC+CA125	0.955 (0.912–0.997)	87.7 (77.9–94.2)	92.5 (79.6–98.4)	81.8 (64.5–93.0)	86.0 (72.1–94.7)	90.0 (73.5–97.9)	0.749	0.892	<0.001
*VC2*									
TEPOC	0.918 (0.872–0.963)	84.0 (77.4–89.2)	86.2 (77.1–92.7)	81.3 (70.7–89.4)	84.3 (75.0–91.1)	83.6 (73.0–91.2)	0.677	0.852	0.033
CA125	0.836 (0.774–0.899)	77.2 (69.9–83.4)	79.3 (69.3–87.3)	74.7 (63.3–84.0)	78.4 (68.4–86.5)	75.7 (64.3–84.9)	0.540	0.789	—
TEPOC+CA125	0.939 (0.901–0.977)	87.7 (81.6–92.3)	89.7 (81.3–95.2)	85.3 (75.3–92.4)	87.6 (79.0–93.7)	87.7 (77.9–94.2)	0.751	0.886	0.002
VC3									
TEPOC	0.887 (0.813–0.960)	82.9 (75.3–89.0)	69.0 (49.2–84.7)	87.0 (78.8–92.9)	60.6 (42.1–77.1)	90.6 (82.9–95.6)	0.534	0.645	0.491
CA125	0.824 (0.663–0.985)	71.4 (53.7–85.4)	70.4 (49.8–86.2)	75.0 (34.9–96.8)	90.5 (69.6–98.8)	42.9 (17.7–71.1)	0.359	0.792	—
TEPOC+CA125	0.917 (0.824–1.000)	74.3 (56.7–87.5)	66.7 (46.0–83.5)	100.0 (63.1–100.0)	100.0 (81.5–100.0)	47.1 (23.0–72.2)	0.478	0.800	0.284

Predictions of TEPOC and the combination model were compared with those of CA125 using a two-sided DeLong’s test (AUC *P*-value). Abbreviations: TEPOC, tumor-educated platelet-derived gene panel of ovarian cancer; CA125, cancer antigen 125; TEPOC+CA125, the combination of TEPOC and CA125; AUC, area under the curve; ACC, accuracy; SN, sensitivity; SP, specificity; PPV, positive predictive value; NPV, negative predictive value; CI, confidence interval.

The AUCs of TEPOC in two Chinese cohorts VC1 and VC2 were 0.923 (95% CI 0.855–0.990) and 0.918 (95% CI 0.872–0.963), respectively. Combining TEPOC with CA125 exhibited an AUC of 0.955 (95% CI 0.912–0.997) in VC1 and 0.939 (95% CI 0.901–0.977) in VC2. Compared with the AUCs of CA125 [VC1, 0.735 (95% CI 0.615–0.855); VC2, 0.836 (95% CI 0.774–0.899)], the AUC increments derived from single TEPOC (VC1, *P* = 0.013; VC2, *P* = 0.033) and its coalition with CA125 (VC1, *P* < 0.001; VC2, *P* = 0.002) were both statistically significant in VC1 and VC2 (Fig. 3B and 3C).

Though developed using Chinese samples, TEPOC demonstrated a diagnostic AUC of 0.887 (95% CI 0.813–0.960) in the European cohort VC3. The AUC of combinatorial diagnosis of TEPOC and CA125 was 0.917 (95% CI 0.824–1.000). Despite the increased AUCs compared with CA125 [0.824 (95% CI 0.663–0.985)], the improvements induced by TEPOC (*P* = 0.491) and the combination (*P* = 0.284) were not significant (Fig. 3D). Therefore, we performed a permutation test in three validation cohorts to prove that the validations were highly unlikely to be false positive (Fig. S8). We also explored the performance of TEPOC to distinguish between benign and malignant adnexal lesions (That is, we used only BAMs as controls). TEPOC performed better than CA125 in the VC1 and VC2, but slightly worse in VC3. Nonetheless, the combination of TEPOC and CA125 still outperforms CA125 alone in identifying OC (Fig. S9). The same cut-off of 0.5 was used to classify the sample as OC across different cohorts and subgroups which was established mainly based on the mean misclassification error in the training cohort (Fig. S10). Other parameters such as accuracy, sensitivity, and specificity are available in Table 2 and the calibration curves are shown in Fig. S11.

### Model performance and its combination with CA125 in ovarian cancer subgroups

**Figure 4.**
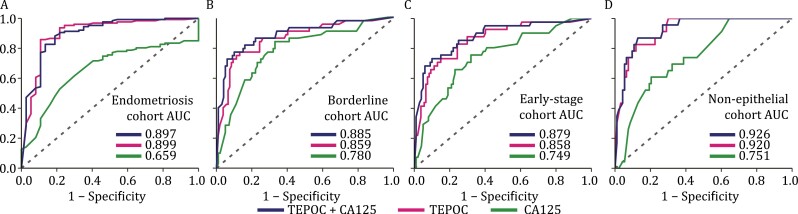
**Performance of TEPOC and its combination with CA125 in ovarian cancer subgroups.** (A) Endometriosis cohort (endometriosis *n* = 37 vs. ovarian cancer *n* = 127), (B) borderline cohort (borderline ovarian tumors *n* = 43 vs. BAM *n* = 107), (C) early-stage cohort (early-stage ovarian cancer *n* = 41 vs. BAM *n* = 107), and (D) non-epithelial cohort (non-epithelial malignancies *n* = 23 vs. BAM *n* = 93). Abbreviations: AUC, area under the curve; TEPOC, tumor-educated platelet-derived gene panel of ovarian cancer; CA125, cancer antigen 125; TEPOC+CA125, a combined diagnosis of TEPOC and CA125; BAM, benign adnexal mass.

We next examined TEPOC in ovarian cancer subgroups. Endometriosis can cause increased plasma CA125 levels and mimic ovarian cancer in ultrasound examinations, making their differential diagnosis a clinical challenge (Giudice, 2010). We included endometriosis (*n* = 37) and ovarian cancer (*n* = 127) in VC1 and VC2 (no endometriosis in VC3) to constitute an endometriosis cohort. The AUC of TEPOC in this cohort [0.899 (95% CI 0.830–0.968)] was significantly higher than that of CA125 [0.659 (95% CI 0.568–0.750), *P* < 0.001; Fig. 4A; Table 3].

**Table 3.** Performance of TEPOC and CA125 to detect ovarian cancer in subgroup analysis.

	AUC (95% CI)	ACC (95% CI), %	SN (95% CI), %	SP (95% CI), %	PPV (95% CI), %	NPV (95% CI), %	Kappa	F1	AUC *P-*value
*Endometriosis*									
TEPOC	0.899 (0.830–0.968)	86.6 (80.4–91.4)	89.0 (82.2–93.8)	78.4 (61.8–90.2)	93.4 (87.4–97.1)	67.4 (51.5–80.9)	0.637	0.911	<0.001
CA125	0.659 (0.568–0.750)	70.1 (62.5–77.0)	80.3 (72.3–86.8)	35.1 (20.2–52.5)	81.0 (73.0–87.4)	34.2 (19.6–51.4)	0.153	0.806	—
TEPOC+CA125	0.897 (0.832–0.963)	87.8 (81.8–92.4)	90.6 (84.1–95.0)	78.4 (61.8–90.2)	93.5 (87.6–97.2)	70.7 (54.5–83.9)	0.664	0.920	<0.001
*Borderline*									
TEPOC	0.859 (0.789–0.929)	76.7 (69.1–83.2)	76.7 (61.4–88.2)	76.6 (67.5–84.3)	56.9 (43.2–69.8)	89.1 (80.9–94.7)	0.483	0.653	0.155
CA125	0.780 (0.696–0.864)	73.4 (65.4–80.5)	74.4 (58.8–86.5)	73.0 (63.2–81.4)	54.2 (40.8–67.3)	86.9 (77.8–93.3)	0.429	0.627	—
TEPOC+CA125	0.885 (0.819–0.951)	83.2 (76.1–88.9)	76.7 (61.4–88.2)	86.0 (77.6–92.1)	70.2 (55.1–82.7)	89.6 (81.7–94.9)	0.611	0.733	0.017
*Early stage*									
TEPOC	0.858 (0.790–0.926)	75.7 (67.9–82.3)	73.2 (57.1–85.8)	76.6 (67.5–84.3)	54.5 (40.6–68.0)	88.2 (79.8–93.9)	0.451	0.625	0.060
CA125	0.749 (0.660–0.839)	70.9 (62.7–78.3)	65.9 (49.4–79.9)	73.0 (63.2–81.4)	50.0 (36.1–63.9)	83.9 (74.5–90.9)	0.355	0.568	—
TEPOC+CA125	0.879 (0.813–0.944)	82.3 (74.9–88.2)	73.2 (57.1–85.8)	86.0 (77.6–92.1)	68.2 (52.4–81.4)	88.7 (80.6–94.2)	0.579	0.706	0.005
*Non-epithelial*									
TEPOC	0.920 (0.868–0.972)	79.3 (70.8–86.3)	82.6 (61.2–95.0)	78.5 (68.8–86.3)	48.7 (32.4–65.2)	94.8 (87.2–98.6)	0.484	0.613	0.002
CA125	0.751 (0.651–0.850)	70.7 (61.5–78.8)	60.9 (38.5–80.3)	73.1 (62.9–81.8)	35.9 (21.2–52.8)	88.3 (79.0–94.5)	0.269	0.452	—
TEPOC+CA125	0.926 (0.875–0.977)	85.3 (77.6–91.2)	87.0 (66.4–97.2)	84.9 (76.0–91.5)	58.8 (40.7–75.4)	96.3 (89.7–99.2)	0.609	0.702	<0.001

Endometriosis cohort (endometriosis *n* = 37 vs. ovarian cancer *n* = 127). Borderline cohort (borderline ovarian tumors *n* = 43 vs. BAM *n* = 107). Early-stage cohort (early-stage ovarian cancer *n* = 41 vs. BAM *n* = 107). Non-epithelial cohort (non-epithelial malignancies *n* = 23 vs. BAM *n* = 93). Predictions of TEPOC and the combination were compared with those of CA125 using a two-sided DeLong’s test. Abbreviations: BAM, benign adnexal mass. TEPOC, tumor-educated platelet-derived gene panel of ovarian cancer; CA125, cancer antigen 125; TEPOC+CA125, the combination of TEPOC and CA125; AUC, area under the curve; ACC, accuracy; SN, sensitivity; SP, specificity; PPV, positive predictive value; NPV, negative predictive value; CI, confidence interval.

Borderline ovarian tumors have malignant potential and are different entities from BAMs in terms of clinical presentation and treatment (Colombo et al., 2019). Borderline ovarian tumors (*n* = 43) and BAMs (*n* = 107) in validation cohorts were aggregated to establish a borderline cohort. Compared with CA125 [0.780 (95% CI 0.696–0.864)], TEPOC achieved a greater [0.859 (95% CI 0.789–0.929)] but not significant (*P* = 0.155) AUC to detect borderline tumors (Fig. 4B; Table 3).

CA125 elevation is typical for advanced ovarian cancer, but lacks accuracy in detecting early-stage ovarian cancer (Zhang et al., 2021). We pooled FIGO stage I-IIA ovarian cancer (*n* = 41) and BAMs (*n* = 107) in three validation cohorts to compose an early-stage cohort. TEPOC exhibited an AUC of 0.858 (95% CI 0.790–0.926) to detect early-stage ovarian cancer, being not significantly different from that of CA125 [0.749 (95% CI 0.660–0.839); *P* = 0.06; Fig. 4C; Table S3).

Since the diagnostic performance of CA125 was unsatisfactory among patients with non-epithelial ovarian malignancies (Zhang et al., 2021), we assessed the efficiency of TEPOC in this subtype. Non-epithelial malignancies (*n* = 23) and BAMs (*n* = 93) in VC1 and VC2 (no non-epithelial malignancies in VC3) were incorporated as a non-epithelial cohort. Compared with CA125 [0.751 (95% CI 0.651–0.850)], TEPOC exhibited a significantly greater AUC to diagnose non-epithelial malignancy [0.920 (95% CI 0.868–0.972), *P* = 0.002; Fig. 4D, Table S3].

Importantly, the combination showed significantly greater AUCs over CA125 to differentiate ovarian cancer from endometriosis [0.897 (95% CI 0.832–0.963) vs. 0.659, *P* < 0.001] and to detect borderline ovarian tumors [0.885 (95% CI 0.819–0.951) vs. 0.780, *P* = 0.017], early-stage ovarian cancer [0.879 (95% CI 0.813–0.944) vs. 0.749, *P* = 0.005], and non-epithelial ovarian malignancies [0.926 (95% CI 0.875–0.977) vs. 0.751, *P* < 0.001] (Fig. 4A–D; Table S3).

High-grade serous disease accounts for over 60% of ovarian cancer (Lheureux et al., 2019a). Using BAMs as controls (*n* = 107), TEPOC displayed an AUC of 0.903 (95% CI 0.856–0.951) to identify high grade serous ovarian cancer (*n* = 82; Table S3). We also analyzed the diagnostic value of TEPOC and CA125 in ovarian cancer subgroups with a pre-specified specificity of 90% (Table S4). The CA125 abundance for ovarian cancer subgroups is provided in Fig. S12. The performance of TEPOC and the combination model (TEPOC+CA125) in detecting ovarian cancer in validation cohorts show that the combination model performed significantly better than TEPOC to detect ovarian cancer only in VC2 (*P* = 0.011, Table S5). We also evaluated the model’s clinical utility using decision curve analysis (Fig. S13). Decision curve analysis indicated that in a wide range of thresholds from 40% to 100%, TEPOC yielded a larger net-benefit vs hypothetical all-detect or no-detect scenarios. Decision curve analysis demonstrated that combined model had a superior net benefit regardless of the probability threshold in the combined cohorts

### Validation for representative contributing genes in the TEPOC model via qPCR

We also performed real-time quantitative PCR (qPCR) to validate some representative genes, which are differentially expressed genes across cohorts in RNA-seq data and contributing genes in the TEPOC model (Supplementary methods). The expression of COL10A1, EIF4G1, VWF was significantly up-regulated in OC compared with non-OC samples, while NPAT was significantly down-regulated in OC compared with non-OC samples (Fig. S14). The primer sequences used in qPCR are shown in the Table S6.

## Discussion

Modes to implement TEPs, the latest components of liquid biopsy, in clinic to unleash their power of cancer detection remain poorly defined. This intercontinental biomarker identification study observed three main findings. First, RNA-sequencing of TEPs enabled accurate preoperative diagnosis of ovarian cancer by demonstrating high AUCs in two Chinese validation cohorts, the European validation cohort, and ovarian cancer subgroups including early-stage disease, non-epithelial malignancies, borderline tumors, and differentiations between ovarian cancer and endometriosis. Second, integrating CA125 with TEPOC significantly enhanced the diagnostic performance of CA125 in four ovarian cancer subgroups and two Chinese validation cohorts.

Besides platelets, ctDNA, CTC or exosomal miRNAs are also potential liquid biopsy methods in OC, which was comprehensively reviewed in a recent paper (Zhu et al., 2022). A recent study of CTC diagnosis model for OC achieved 79.4% and 92.2% in sensitivity and specificity, respectively (Wang et al., 2022). The AUC of a published model with three exosomal miRNAs for OC diagnosis was 0.8337 (Chen et al., 2022). A French group reported that the plasma sequencing can detect ctDNA 88% OC cases (Sabatier et al., 2022). Comparing with these models, the performance of our TEPOC is higher or comparable. However, other diagnostic models in liquid biopsy, such as ctDNA, CTC or exosomal miRNAs still face technical challenging due to lower concentrations and high testing cost. Platelets, as the second-most abundant cell type in peripheral blood, are easily isolated, contributing to the simplicity of sample processing methods. The large number of platelets in peripheral blood and their interaction with tumour cells collectively facilitate the development of a cost-effective diagnostic model which could serve as promising complement for traditional liquid biopsy (Zhu et al., 2022). Here, we firstly provide the evidence for potentially effective applications of TEPs for preoperative ovarian cancer diagnosis. Besides its practicability, TEPOC displays robustness across different ethnicities, heterogenous subtypes of ovarian cancer. Though developed using platelet RNAs derived from Chinese population, TEPOC yielded an AUC of over 0.88 in the European cohort, despite the racial differences of gene expressions within platelets (Edelstein et al., 2013). Unfortunately, the superiority of TEPOC or the combination over CA125 was not significant in the European cohort, which could be attributable to the much lower proportion of OC, the older median age of participants, insufficient sample size, less sequencing reads, and racial differences.

Endometriosis can cause ovarian cysts and may increase CA125 levels (Giudice, 2010). Individuals with indeterminate ovarian cysts and concomitant increased CA125 levels are readily suspected to be afflicted with ovarian cancer by clinicians, leading to tremendous anxiety and subsequent surgery that can even impair ovarian function. This study suggested that the demerit of CA125 might be mitigated by introducing TEPOC. Accurate detection of early-stage curable ovarian cancer is an essential factor that influences ovarian cancer prognosis (Vaughan et al., 2011). Whether TEPOC can contribute to the early detection or screening of ovarian cancer needs to be investigated in a larger population. Though only 20% of ovarian tumors are non-epithelial (Prat and Mutch, 2018), they constitute a diagnostic challenge. TEPOC allowed more accurate detection of non-epithelial ovarian malignancies than CA125. Numerous combinatorial strategies have been investigated to improve the diagnostic accuracy of CA125, including a combination of CA125 and sequential transvaginal ultrasound. Integrating CA125 with TEPOC significantly improved the performance of CA125, suggesting that TEPOC might be an effective combinatory biomarker for CA125.

It would make sense to evaluate diagnostic performance of TEPOC in BRCA mutant high-risk OC patients. However, with the launch of Olaparib in China in August 2018, BRCA testing began to be carried out on a large scale in China. The number of samples collected during this period contained BRCA information was small, so it was impossible to accurately evaluate the prediction performance of the model in BRCA mutant OC patients. We do have a plan to further test diagnostic performance of TEPOC in BRCA mutant OC patients through prospective clinical trial.

The major strength of the present study was the robustness of TEPOC, which could be validated in individuals of different ethnicities, heterogenous subtypes of ovarian cancer. From a technical aspect, we adopted MRGF algorithm that enables dimensionality reduction to select contributing genes, which properly leverages the high-dimensional and small-sample-size RNA sequencing data. LASSO and mRMR methods were combined to yield minimum but stable differential RNA profile since LASSO could largely reduce the number of non-relevant genes and mRMR could rank the contribution of genes. However, this study has some limitations to address. First, TEPOC was developed using diagnostic samples when the tumor becomes clinically apparent. The findings warrant prospective investigations in population-based pre-diagnostic and early-stage ovarian cancer samples. Second, the sample size of early-stage ovarian cancer and non-epithelial ovarian malignancies were insufficient due to their relatively low incidence. Third, the incapability to analyze the performance of human epididymis protein 4 (HE4) or its combination with CA125 resulted from the low detection rate, lack of standardization, and variations of HE4 tests in China. Finally, although the number of genes in TEPOC was quite small compared with existing studies based on large sample platelet transcriptome sequencing (Best et al., 2017; In ‘t Veld et al., 2022), we believe the number could be further reduced in the future study. To circumvent these limitations, population-based prospective validations in more patients in ovarian cancer subgroups and pan-cancer analysis are necessary to consolidate our findings.

## Materials and methods

### Multicentric, real-world data collection of platelet count

The inclusion and exclusion criteria for the real-world paraneoplastic thrombocytosis exploration in ovarian cancer are as follows: The inclusion criteria: individuals with platelet counts recorded within seven days before administration of any tumor-related treatment; ovarian cancer included primary ovarian cancer, fallopian tube cancer, or peritoneal carcinoma [International Classification of Diseases-10 (ICD-10): C56.x00, C57.801, and C57.000)]; the controls included healthy individuals and patients with benign lesions on ovaries, fallopian tubes, or uterus. The exclusion criteria: having a history of other malignancies or precancers other than ovarian cancer; in pregnancy in the last six months; having HIV infection; not newly diagnosed. Recurrent ovarian cancer cases were regarded as newly diagnosed if no antitumor treatment was introduced. Platelet counts of 1,301 naive-treatment patients with ovarian cancer (OC) and 8,667 benign adnexal masses (non-OC) individuals were consecutively collected from three domestic hospitals between January 2013 and August 2016.

### Processing of blood samples and raw RNA-sequencing data

Pre-treatment blood samples retrieved from all medical centers were processed using the same standardized protocols as the previous literature described Best et al., (2019) and detailed in Supplemental Methods. The platelet separation method ensured the purity of platelets (Fig. S1) and was confirmed not to cause platelet activation within 48h (Fig. S2). Total RNA was extracted from platelets and sequenced on the HiSeq (2500/4000) or HiSeq X-ten platform (BGI-Shenzhen, China) using the standard protocol elaborated in Supplemental Methods. The raw data of platelet RNA-sequencing in FASTQ files were subjected to our in-house RNA-sequencing process pipeline. Briefly, raw reads were trimmed using FastQC (v0.11.8) and clean reads were mapped to human reference genome (hg19) by STAR (v2.7.0) (Dobin et al., 2013). Aligned reads were subjected to HTSeq 2 with Ensembl gene annotation version 87 to quantify gene expression (Anders et al., 2015; Yates et al., 2020). Samples with total read count less than 5 × 10^6^ were filtered. Genes with < 10 reads in over 10% of cohort samples and hypervariable genes (coefficient of variation > 3) were excluded by R-package ineq (v0.2.13).

### Clustering of discovery cohort

Filtering yielded genes with low inequality and sufficient coverage in discovery cohort. The filtered read count matrix of these genes was normalized via variance stabilizing transformation with the software program DESeq2 (v1.26.0), and differentially expressed genes (DEGs) were determined on the basis of the apeglm log2 fold-change shrinkage algorithm in DESeq2 (cut-off: adjusted *P* < 0.05 and base mean > 10) (Love et al., 2014; Zhu et al., 2019). Finally, discovery cohort was subjected to clustering guided by these DEGs through unsupervised hierarchical clustering using Complex Heatmap (v2.2.0) using Ward’s clustering and Pearson’s correlation distances (Gu et al., 2016).

### Study design and participants

Inclusion and exclusion criteria of the diagnostic study are detailed below: Inclusions: 1) Patients having various types of adnexal masses with definite histological diagnosis and healthy women with no adnexal lesions found in regular physical examination willing to participate in the clinical study; 2) Patients with adnexal mass without any treatment before blood collection; 3) Patients with malignant adnexal lesions had no history of malignancy other than ovarian cancer and patients with non-malignant adnexal lesions had no history of malignancy; 4) Participants with qualified platelet samples [RNA integrity number < 7 and quantity < 10 picogram of total RNA]. Exclusions: 1) Patients with adnexal mass received any treatment before blood collection; 2) Patients with malignant adnexal lesions had malignancy other than ovarian cancer and patients with non-malignant adnexal lesions had any malignancy; 3) Participants with any underlying disease that affects platelet production and apoptosis; 4) Participants with unqualified platelet samples [low quality (RNA integrity number < 7) or quantity (< 10 picogram) of total RNA].

In this intercontinental collaboration, treatment-naïve blood samples were obtained from consecutive inpatients with adnexal masses and random healthy women controls in nine medical centers (China, *n* = 3; Netherlands, *n* = 5; Poland, *n* = 1) between September 2016 and May 2019. Participants constituted five independent cohorts including a discovery cohort, a training cohort, two Chinese (VC1 and VC2) validation cohorts, and a European validation cohort (VC3). Participants in discovery cohort and training cohort were from the Department of Gynecologic Oncology of Tongji Hospital. Participants in VC1 and VC2 were from the Department of Gynecologic Oncology of Union Hospital and Guanggu Hospital, respectively. Three Chinese hospitals were all affiliated to Tongji Medical College, Huazhong University of Science and Technology. VC3 comprised participants from five medical centers in the Netherlands (Utrecht University Medical Center; Amsterdam University Medical Center, VU University Medical Center; Amsterdam University Medical Center, Amsterdam Medical Center; Leiden University Medical Center; Catharina Hospital) and one center in the Poland (The Medical University of Gdańsk).

This study was approved by the appropriate ethical committees (Supplemental Methods), performed in compliance with the principles of Declaration of Helsinki, registered at Chinese Clinical Trial Registry as #ChiCTR2100046452, and reported following Standards for Reporting Diagnostic accuracy studies (STARD) guidelines.

All consecutive inpatients with adnexal masses were histologically confirmed without the knowledge of the CA125 concentration or the judgment of TEPs. The reference standard was the histological diagnosis based upon the 2014 World Health Organization classification for ovarian tumors (Longacre et al., 2014). International Federation of Gynecology and Obstetrics (FIGO) stages I and stage IIA ovarian cancers were classified as early-stage, whereas FIGO stages IIB to IV malignancies were considered late-stage (Prat, 2014). Healthy women did not have a history of malignancy or adnexal-related lesions. Patients with BAMs and healthy women were referred to as individuals without cancer. Clinical data related to histological subtypes, FIGO stages, and tumor grades were retrieved from the electronic health records, proofread by two investigators independently (YG and CJL), and reviewed by the third researcher (XMX). Pre-treatment CA125 tests were performed prior to blood sampling.

### Data normalization and correction of confounding variables

The procedures are elaborated in Supplemental Methods. To eliminate the influence of batch effects and confounding variables in the classification model, we performed routine data processing in RNA-sequencing data mining (Chiesa et al., 2018). Variance stabilizing transformation in DESeq2 was used to normalize the read count data and investigate the correlation between confounding and surrogate variables generated via svaseq (Leek, 2014).

### Feature selection and model construction

We designed a minimum redundant gene filtering (MRGF) algorithm for high dimensional RNA-sequencing data feature selection and performed it based on sequencing data in training cohort to build models. Briefly, we first excluded genes with low abundance and high inequality. Second, we excluded genes with high internal correlation via the findCorrelation function in the R-package caret (Kuhn, 2008). Third, we adopted the least absolute shrinkage and selection operator (LASSO) approach (Tibshirani, 1996). The regularization path was determined for the LASSO penalty at the grid of values for regularizing the lambda parameter using the R-package glmnet and caret with 10-fold cross-validation (Friedman et al., 2010). We selected genes that contributed to the final model with the best-tuned lambda value. Fourth, to identify robust and optimal features, we used minimum redundancy maximum relevance (mRMR) for further selection (Ding and Peng, 2005). The mRMR.ensemble function in the R-package mRMR was used to rank features with relevant scores, followed by increment feature selection in a 10-fold cross-validation support vector machine (SVM) with default hyperparameter tuning. The final RNA profile yielded sufficient diagnostic performance and expected number of genes. Fifth, we optimized the SVM hyperparameters cost and sigma through 5000 random searches, with 10-fold cross-validation. Finally, we trained the SVM model with the RNA profile and optimized the cost and sigma parameters.

### Statistical analysis

The sample size estimation was performed using NCSS PASS (version 11.0.10) and depicted in Supplemental Methods. The main outcomes were the diagnostic performance (area under the receiver operating characteristics curve, AUC) of TEPs and its combination with CA125 in validation cohorts. Secondary analysis was conducted in ovarian cancer subgroups including early-stage ovarian cancer, borderline ovarian tumors, non-epithelial ovarian malignancies, and differential diagnosis between ovarian cancer and endometriosis. The AUCs and 95% confidence intervals (CI) were calculated, and other metrics (accuracy, Kappa, and F1) were determined using the R-package epiR. Training, tuning, and prediction were performed using the R-package mlr. Predictions generated by the classifier were compared with the results of CA125. All Figs herein without specification were plotted with the R-package ggplot2. The performance of detection test *P*-values was calculated via a two-sided DeLong’s test. The *P*-values of other tests without specification were calculated via a two-sided Student’s *t*-test. The probability was then used to determine the classification of the test result. *P*-values less than 0.05 were considered statistically significant. Other method descriptions are detailed in supplementary methods.

## Conclusion

In this retrospective, intercontinental biomarker identification study, we developed and validated a TEPs-derived RNA signature that enabled early, accurate, and non-invasive detection of ovarian cancer. It had robustness, compatibility, and universality in patients of different ethnicities, heterogenous histological subtypes of ovarian cancer. However, these observations warrant prospective validations in a larger population before clinical utilities.

## Supplementary information

The online version contains supplementary material available at https://doi.org/10.1093/procel/pwac056.

pwac056_suppl_Supplementary_Material

## Data Availability

Data will be made available on execution of appropriate Material Transfer Agreement via qingleigao@hotmail.com.

## Abbreviations

ACC: accuracy
AUC: area under the curve
BAM: benign adnexal mass
CA125: cancer antigen 125
CI: confidence interval
CTCs: circulating tumor cells
ctDNA: circulating tumor DNA
CV: cross-validation
DC: discovery cohort
DEGs: differentially expressed genes
FIGO: International Federation of Gynecology and Obstetrics
IFS: increment feature selection
IQR: interquartile range
LASSO: least absolute shrinkage and selection operator
MRGF: minimum redundant gene filtering (MRGF)
mRMR: minimum redundancy maximum relevance
NPV: negative predictive value
OC: ovarian cancer
PCA: principal component analysis
PGF: placental growth factor
PPV: positive predictive value
qPCR: real-time quantitative PCR
SN: sensitivity
SP: specificity
SVM: support vector machine
TEPOC: TEP-derived gene panel of ovarian cancer
TEPs: tumor-educated platelets
TC: training cohort
VC1: validation cohort 1
VC2: validation cohort 2
VC3: validation cohort 3
