# Supplementary material for: Platelet RNA enables accurate detection of ovarian cancer: an intercontinental, biomarker identification study
Source: Protein Cell. 2022 Nov 10;14(8):579–90. doi: 10.1093/procel/pwac056 (PMC10246718; doi:10.1093/procel/pwac056)
Supplement: pwac056_suppl_Supplementary_Material [file pwac056_suppl_supplementary_material.pdf]

Supplementary Methods

Ethical Approval

This study was approved by the Ethical Committee of Tongji Medical College, Huazhong University of Science and Technology (S253) and the Ethical Committee of the Leiden University Medical Center (#P16.229). Sample collection in Gdańsk (Poland) was approved by the Independent Ethics Committee of the Medical University of Gdańsk (NKBBN/434/2017). Sample collection at the Amsterdam University Medical Center was approved by the local Medical Ethics Committee of the hospitals. Sample collection from Catharina hospital was approved by the medical research ethics committees united (W16.063).

Total RNA extraction and RNA-sequencing library preparation

Platelet isolation and RNA extraction for samples derived from nine medical centers were conducted using the same protocol.[1, 2] Strictly same sample processing and storage procedures before sequencing were followed in the central laboratories in China, the Netherlands, and the Poland to preclude the regional differences that may influence the classification performance. Peripheral venous blood was drawn from treatment-naïve participants in 6mL purple-capped BD Vacutainers containing the EDTA anticoagulant. All blood samples were processed within 48 hours of sampling. Specifically, platelets were isolated from whole blood using a standard gradient centrifugation method. The resulting platelet pellets were gently resuspended in RNAlater (Thermo Fisher Scientific, Waltham, MA, USA) and incubated at 4℃ overnight and then transferred to −80℃ after sharp freezing overnight in liquid nitrogen until being sequenced. The platelet separation method ensured the purity of platelets (Supplementary Figure S2) and was confirmed not to have caused platelet activation (Supplementary Figure S3). Total RNA of samples with low quality (RNA integrity number $<$ 7) or quantity ($<$ 10 picogram) were excluded.

For samples with total RNA $\geq$ 50 nanogram, total RNA was extracted from the platelets using TRIzol reagent (Invitrogen; Thermo Fisher Scientific, Inc.) in accordance with the manufacturer’s instructions. The mix was centrifuged at 12 000 × g for 5 min at 4℃. The

supernatant was transferred into a new Eppendorf tube with 0.3 mL chloroform/isoamyl alcohol (24:1). The mix was shaken vigorously for 15 s and then centrifuged at 12 000 × g for 10 min at 4°C. The upper aqueous phase containing RNA was transferred into a new tube with an equal volume of isopropyl alcohol and centrifuged at 12 000 × g for 20 min at 4°C. After discarding the supernatant, the RNA pellet was washed twice with 1 mL 75% ethanol, and the mix was centrifuged at 12 000 × g for 3 min at 4°C to collect residual ethanol, followed by air-drying of the pellet for 5–10 min in the biosafety cabinet. Finally, 25~100 μL of DEPC-treated water was added to dissolve the RNA pellet. Subsequently, total RNA was qualified and quantified using a Nano Drop spectrophotometer and an Agilent 2100 bioanalyzer (Thermo Fisher Scientific, MA, USA).

For samples with total RNA < 50 nanogram, total RNA was extracted from platelets using the RNeasy Micro Kit (QIAGEN, 74004) in accordance with the manufacturer's instructions. Appropriate platelets were ground to powder with liquid nitrogen and then transferred into a new tube with an appropriate volume of Buffer RL and 1 volume 70% ethanol. The mixture was transferred into a RNeasy MinElute spin column and centrifuged at ≥ 8000 × g for 15 s. After discarding the flow-through, Buffer RW1, DNase I, Buffer RPE, and 80% ethanol were added and then sequentially centrifuged. The RNeasy MinElute spin column containing RNA was placed in a new 2-mL collection tube and centrifuged with lid opened at 12 000 × g for 5 min to dry the membrane and then transferred to a new 1.5-mL tube with 14 μL RNase-free water. Finally, the tubes were centrifuged for 1 min at 12 000 × g to elute the RNA. Total RNA was qualified and quantified using a Nano Drop and Agilent 2100 bioanalyzer (Thermo Fisher Scientific, MA, USA).

For samples in the discovery cohort, DNase I was used to digest double- and single-strand DNA in total RNA. Thereafter, magnetic beads were purified to recover the reaction products. The RNase Hor Ribo-Zero method (human, mouse, plants) (Illumina, San Diego, CA, USA) was used to eliminate rRNA. Purified mRNA was fragmented into small pieces using fragment buffer. Thereafter, the first-strand cDNA was generated in the First Strand Reaction System via PCR, and the second strand of cDNA was also generated. The reaction product was purified using magnetic beads. A-Tailing Mix and RNA Index Adapters were added for

end repair. The cDNA fragments with adapters were amplified via PCR and the products were purified via Ampure XP Beads. The quality and quantity of the library were assessed via two methods to ensure the high quality of the sequencing data: one method involved assessing the distribution of the fragment sizes using the Agilent 2100 bioanalyzer; the other method involved quantifying the library via real-time quantitative PCR. The qualified library was amplified on cBot to generate the cluster on the flowcell, and the amplified flowcell would be sequenced single-end on the HiSeq4000 platform.

For samples with total RNA > 50 nanogram, except those in the discovery cohort, oligo(dT)-attached magnetic beads were used to purify mRNA. Purified mRNA was fragmented with fragment buffer at 94℃ for 5min. Thereafter, the first strand of cDNA was generated using the First Strand reaction system via PCR and then the second strand of cDNA was generated. The reaction product was purified using Ampure XP Beads and dissolved in EB solution. The quality and quantity of the library were assessed via two methods to ensure the high quality of the sequencing data: one method involved assessing the distribution of the fragment sizes using the Agilent 2100 bioanalyzer; the other method involved quantifying the library via real-time quantitative PCR. The qualified library was amplified on cBot to generate the cluster on the flowcell. Moreover, the amplified flowcell will be sequenced single-end on the HiSeq4000 or HiSeq X-ten platform (BGI-Shenzhen, China).

For samples with total RNA between 10 picogram and 50 nanogram, the platelet RNA was amplified with oligo-dT and dNTPs, incubated at 72℃, and immediately placed on ice, followed by reverse transcription to form cDNA, based on the polyA tail method. The template was switched to the 5′ end of the RNA, and full-length cDNA was generated via PCR. The Agilent 2100 bioanalyzer instrument (Agilent High Sensitivity DNA Reagents) was used to determine the average molecule length of the PCR product. The cDNA library was quantified using the Qubit dsDNA HS Assay Kit (Thermo Fisher Scientific, Waltham, MA, USA) for accurate quantification, followed by fragmentation with fragment buffer. Thereafter, the A-Tailing Mix and RNA Index Adapters were added for end repair. The cDNA fragments with adapters were amplified via PCR. The PCR products were purified using Ampure XP Beads and then were size-selected. The final library was quantitated using two methods to

ensure the high quality of the sequencing data: one method involved determining the average molecule length by using the Agilent 2100 bioanalyzer instrument (Agilent DNA 12000 Reagents); the other method involved quantifying the library via real-time quantitative PCR (qPCR). The qualified libraries were amplified using cBot to generate the cluster on the flowcell. The amplified flowcell was sequenced single-end on the HiSeq4000 platform (BGI-Shenzhen, China).

Data normalization and batch effect removal

In the normalization process, raw read counts of training cohort were subjected to "Variance Stabilizing Transformation" with parameter "blind=FALSE" for normalization and "Dispersion Function" for dispersion estimation by using R-package DESeq2.[3] For the validation cohorts, we assigned the estimated dispersion values from the training cohort as their dispersion and used the same method to normalize them. To exclude samples with low inter-sample correlation, we used the "Bigcor" function of R-package propagate to perform Pearson correlation, yielded one sample with a correlation of < 0.4, which was excluded from the training cohort.

To minimize the influences of age (Supplementary Figure S6A), library size (Supplementary Figure 6B), and known batches for further classification, we investigated these potential confounding factors with surrogate variables identified via svaseq in R-package sva with default parameters.[4] Each estimated surrogate variable was correlated with the potential confounding factors in cancer or non-cancer group. The continuous variables were correlated to surrogate variables by Pearson correlation and categorical variables were compared using a two-sided Student's *t*-test. To prevent eliminating a surrogate variable probably correlated with the cancer or non-cancer group, the surrogate variables with a correlation *P*-value < 0.05 would not be adjusted. These identified confounding factors were used to adjust the normalized data by removeBatchEffect from the R-package limma.[5] The *P*-values between confounding factors and surrogate variables are illustrated in Supplementary Figure S6C. We compared the performance before and after eliminating confounding factors and plotted the relative log intensity (RLE) using the plotRLE function in the R-package EDASeq (Supplementary Figure S6D).

Detailed model development procedure

Four steps were applied to select genes and finally trained SVM model as described in Figure S4. In the classifier development based on RNA-Seq data, which contains small samples and many features (over 60,000 genes), conventional approach was using differential expression genes to select genes between tumour and non-tumour with hand-coded fold change > 2 and FDR < 0.05 [6]. We filtered low abundant and hypervariable genes with mapping reads and expression inqualilty. LASSO was only used to select contributing genes [7] between tumor and non-tumor to reduce high dimension as you acknowledged in the following comment. For further application of our TEPOC model, we tried to eliminate the number of genes in the model. MRMR was used to rank the genes and balance the number of genes and AUC performance [8]. Finally, the optimized number of genes was used to train the SVM model.

Sample size estimation

The sample size calculation was based on the following assumptions. According to the previous hospitalized patients in the Department of Obstetrics and Gynecology of Tongji Hospital, the ratio of ovarian cancer to non-cancer is about 0.8 (231:289) in the training cohort. We designed to achieve the superiority of tumor-educated platelets (AUC=0.9) over CA125 (AUC=0.8). Using a two-sided chi-square test, 80% power would be achieved on the two-sided significance level α=0.2. The minimum sample size was 66 (40 for ovarian cancer and 26 for case control). It was planned to include 74 patients in the validation cohort assuming a dropout rate of 10%. All participants that met the inclusion criteria would be consecutively enrolled until all cohorts reached the minimum sample size.

Validation method for Quantitative real-time (qPCR)

Total RNA was extracted using TRIzol reagent (Invitrogen, Thermo Fisher Scientific, Inc.) in accordance with standard manufacturer's protocols. qPCR was

performed in triplicate (n = 3) using the Bio-Rad CFX96 system with SYBR Green Supermix. The relative mRNA expression levels were calculated using the comparative Cq method $2^{-\Delta\Delta}$Cq) on the basis of ACTB as the loading control.

Statistical analysis

The F1-score combines the precision and recall of a classifier into a single metric by taking their harmonic mean. It is primarily used to compare the performance of two classifiers. The formula for the F1 score is: F1 = 2 * (precision * recall) / (precision + recall). Permutation test is a popular technique for testing a hypothesis of no effect, when the distribution of the test statistic is unknown. We permutated patient label with 5000 times to generate a random AUC distribution to test the p-value of our TEPOC AUC [9].

Supplementary Figures

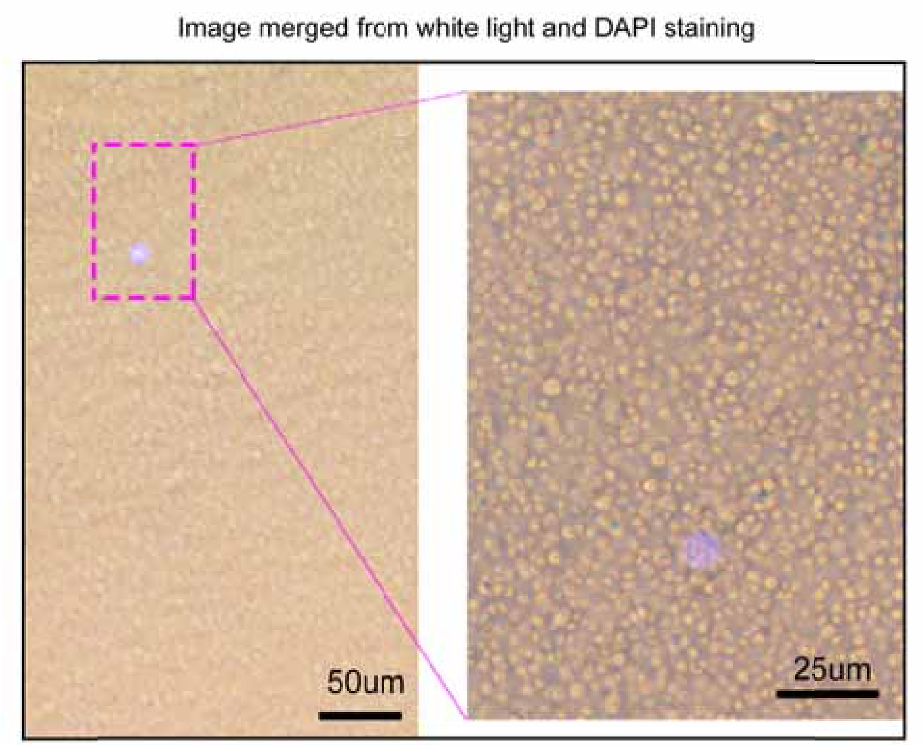

Figure S1. The representative graph of quality control for platelet purity. We adopted the gradient centrifugation to isolate platelets based on previous literature,[10] and assessed platelet purity for all samples by fixing platelet isolations (in RNAlater) in 3.7% paraformaldehyde and staining using DAPI. Total platelet and nucleated cell counts were determined by manual cell counting in 5 μL cell counting chambers on the fluorescence microscope and yielded an estimated 1 to 5 nucleated cell counts per 10 million platelets, which was consistent with the observations by others.[10] Nucleated cells were stained with blue fluorescence (DAPI staining).

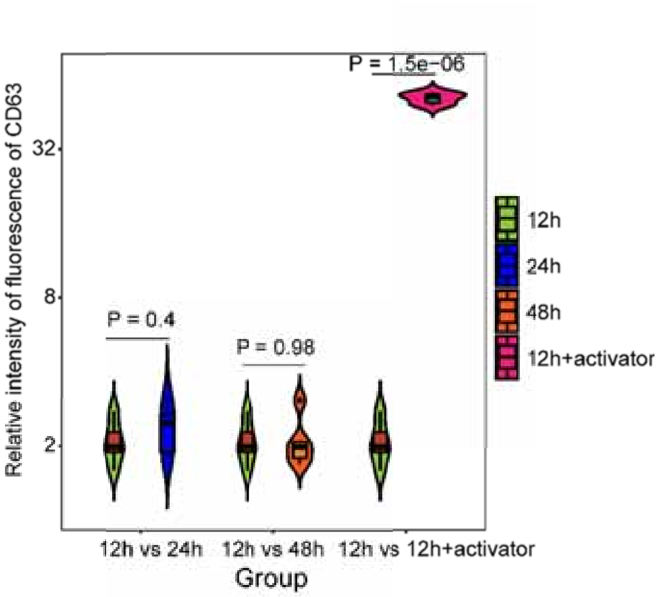

Figure S2. Flow cytometric platelet activation analysis. Box and jitter plots violin plots showing relative levels of CD63 across platelets isolated at different time points. To assess the relative platelet activation during sample processing, we measured the platelet activation-dependent marker CD63 (Biolegend) using a BD FACSCalibur flow cytometer. Four 6-mL EDTA-coated blood were collected from healthy donors, and the platelet activation state was determined at 12 hours, 24 hours, and 48 hours. As a negative control, we isolated at time 12 hours platelets from whole blood using a standardized platelet isolation protocol from whole blood that has been validated for inducing minimal platelet activation.[10] As a positive control, we included platelets activated by prothrombin (Sigma-Aldrich, 1 unit per mL). Platelet pellets after isolation were prefixed in 0.5% formaldehyde (Roth) for flow cytometric analysis. Relative activation and mean fluorescent intensity values were assessed. Stable levels of CD63 from samples of 24 hours and 48 hours suggested there was no platelet activation during blood collection and storage. Kruskal-Wallis tests were performed using R (version 3.5.3). Distributions were plotted with R as violin plot graphs.

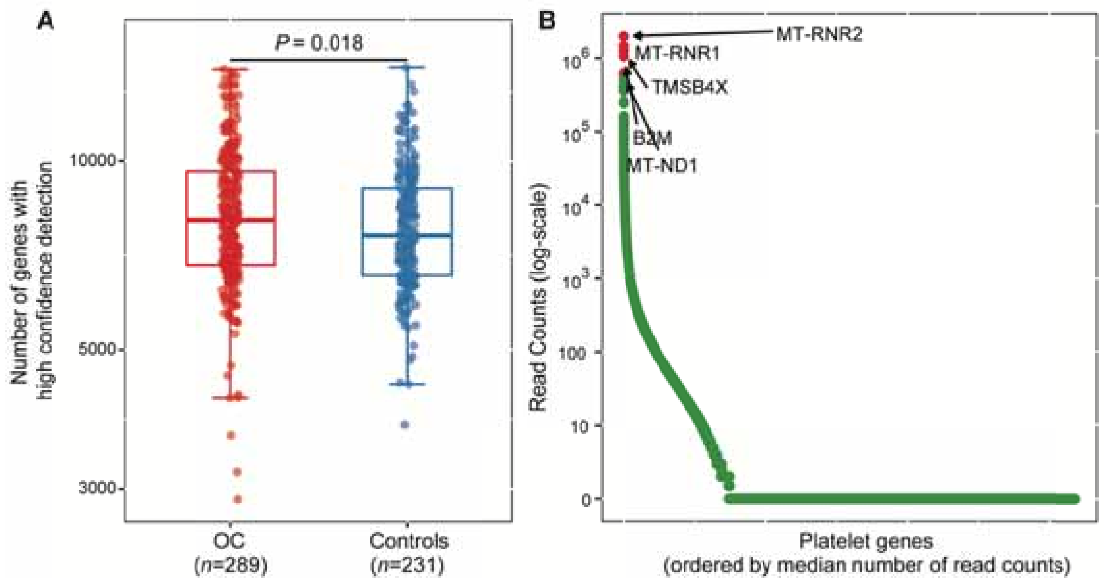

Figure S3. Analysis of the platelet RNA repertoire in training cohort. (**A**) The difference in number of genes with high confidence (over 30 reads) between OC and controls in training cohort. Boxplots represent median value, with lower and upper hinges corresponding to the 25th and 75th percentiles, and lower and upper whiskers extending from the hinge to the smallest and largest value at most 1.5× interquartile range of the hinge, respectively. Two-sided Student's *t*-test. (**B**) Median total read counts of each gene in the training cohort. Five genes with the highest expression are MT-RNR2, MT-RNR1, TMSB4X, B2M, and MTND1. OC, ovarian cancer. Controls included patients with benign adnexal masses and healthy women.

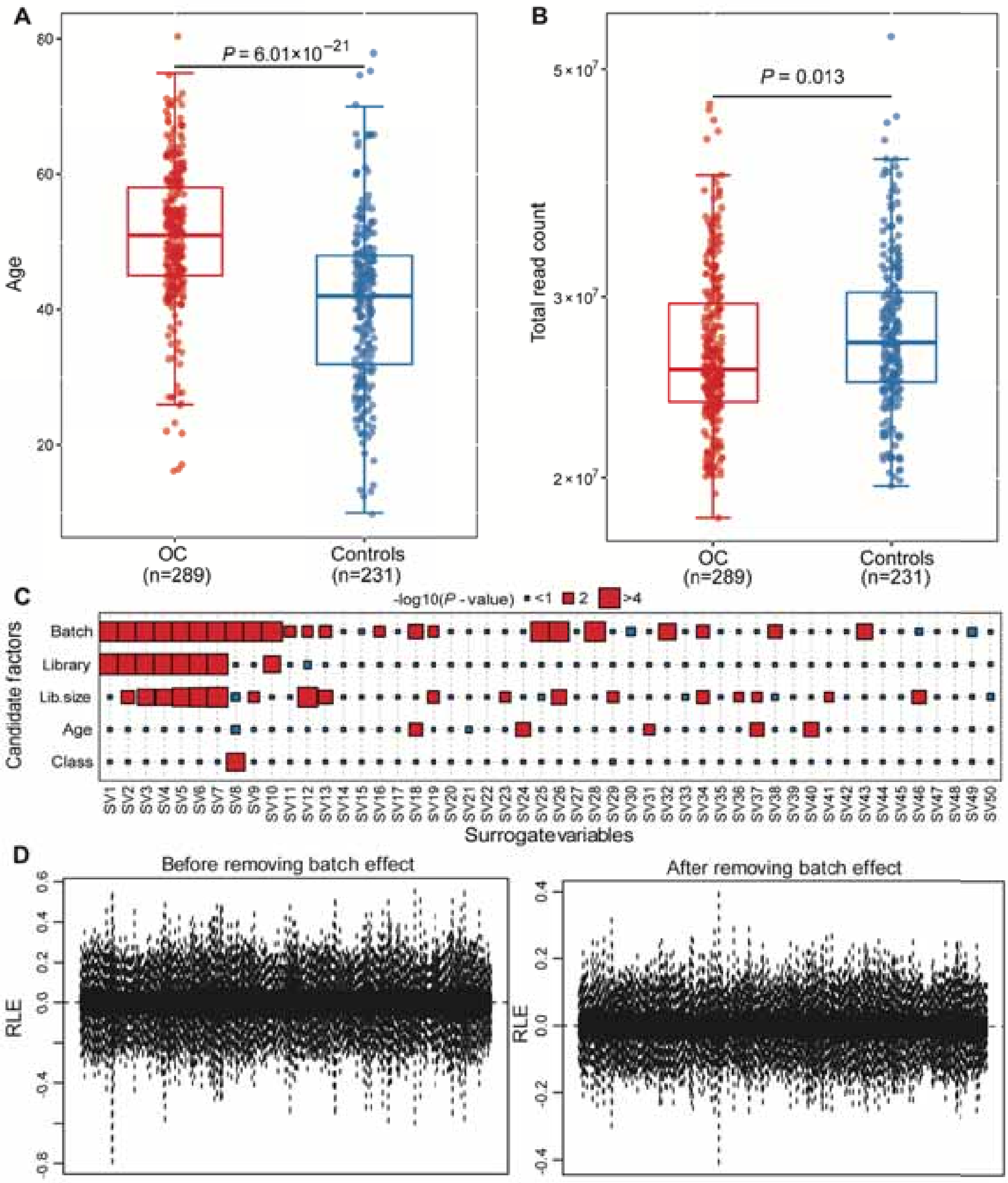

Figure S4. Data normalization and adjustment of confounding variables. (**A**) The age of OC group and controls. (**B**) The library size of OC and control samples. (**C**) The *P*-values between confounding factors and surrogate variables were illustrated. Correction of surrogate variables with candidate confounding factors including batch effect, library, library size, and age. (**D**) Relative log expression (RLE) before (left) and after (right) removal of batch effect. For (**A**) and (**B**), boxplots represent median value, with lower and upper hinges corresponding to the 25th and 75th percentiles, and lower and upper whiskers extending from the hinge to the smallest and largest value at most 1.5× interquartile range of the hinge, respectively. Two-sided Student's *t*-test. OC, ovarian cancer. Controls included patients with benign adnexal masses and healthy women.

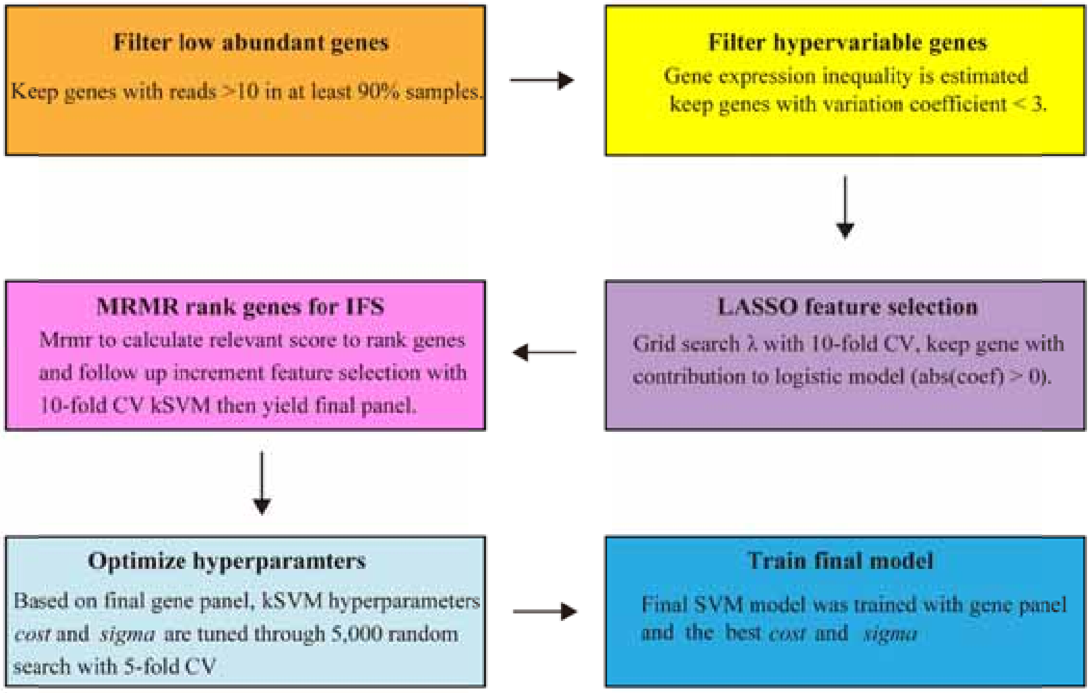

Figure S5. Schema of MRGF and model construction. MRGF, minimum redundant gene filtering. CV, cross-validation. IFS, increment feature selection. SVM, support vector machine. LASSO, least absolute shrinkage and selection operator. MRMR, minimum redundancy maximum relevance.

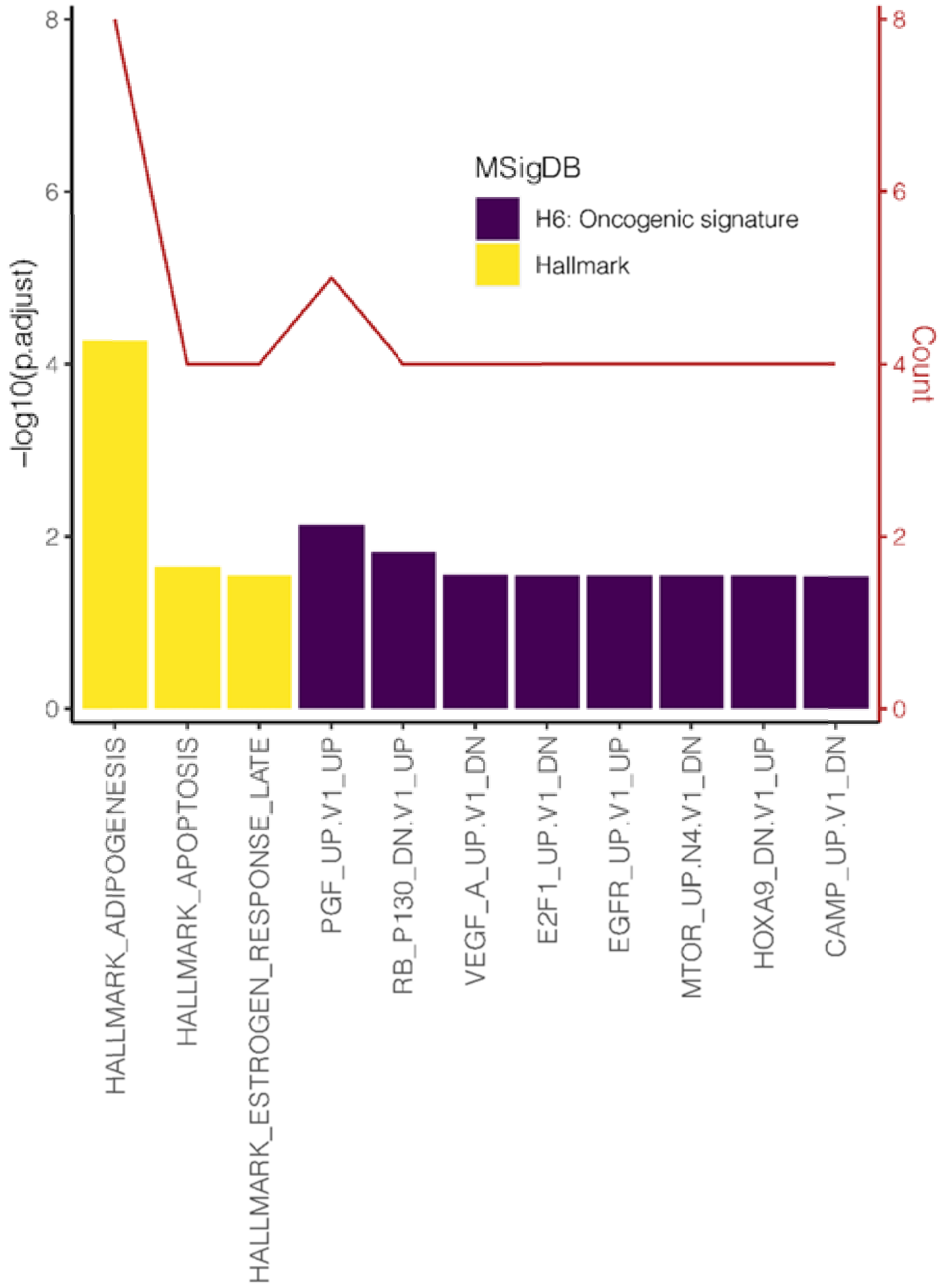

Figure S6. Enrichment analysis of the 102 contributing genes of TEPOC. The gene set (Supplementary Table S2) was enriched based MSigDB cancer hallmark gene set collection (in yellow) and C6 oncogenic signature gene set collection (in purple).

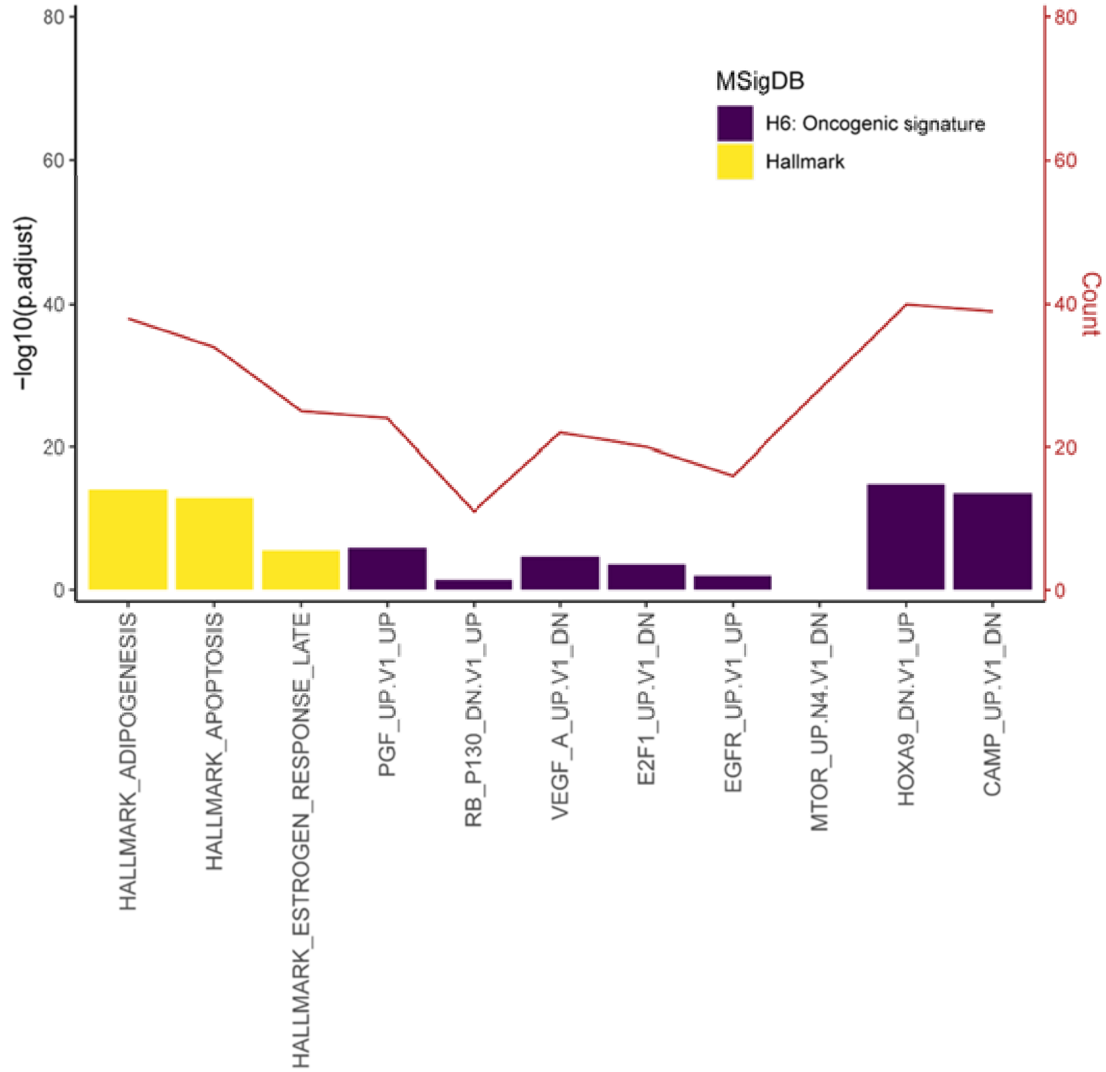

Figure S7. Enrichment analysis of the 1625 differentially expressed genes of lung cancer diagnosis paper. The gene set was enriched based MSigDB cancer hallmark gene set collection (in yellow) and C6 oncogenic signature gene set collection (in purple).

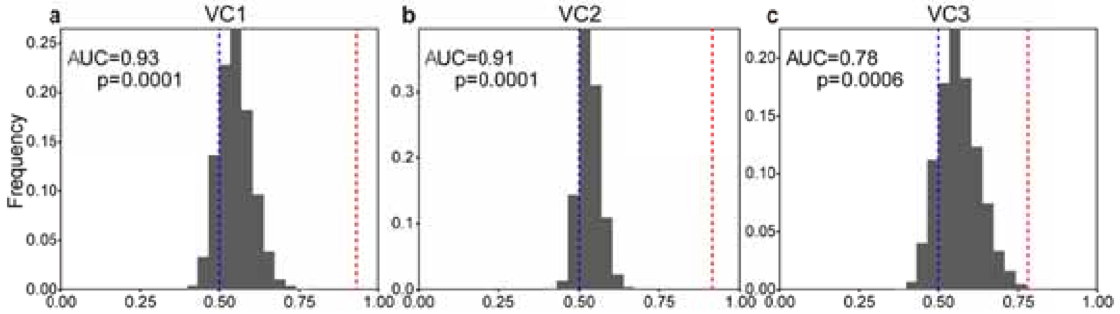

Figure S8. 5,000 simulations for three validation cohorts. Simulations in VC1 (**A**), VC2 (**B**), and VC3 (**C**). Blue vertical line is 0.5 of AUC and red vertical line is the model predicted AUCs. VC, validation cohort. AUC, area under the curve.

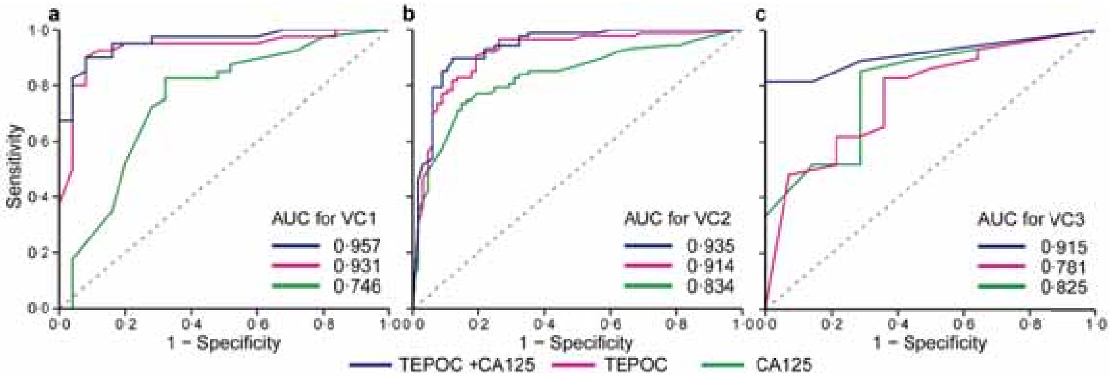

Figure S9. Performance of TEPOC to detect ovarian cancer among patients with adnexal lesions in validation cohorts. Performance of CA125 (green line), TEPOC (red line), and TEPOC+CA125 (blue line) to detect ovarian cancer among patients with adnexal lesions in validation cohort 1 (malignant, n=40; benign, n=25) (a), validation cohort 2 (malignant, n=87; benign, n=68) (b), and validation cohort 3 (malignant, n=29; benign, n=15) (c) using receiver operating characteristic (ROC) curves. AUC, area under the ROC curve. TEPOC, tumour-educated platelet-derived gene panel of ovarian cancer. CA125, carbohydrate antigen 125. TEPOC+CA125, a combined diagnosis of TEPOC and CA125.

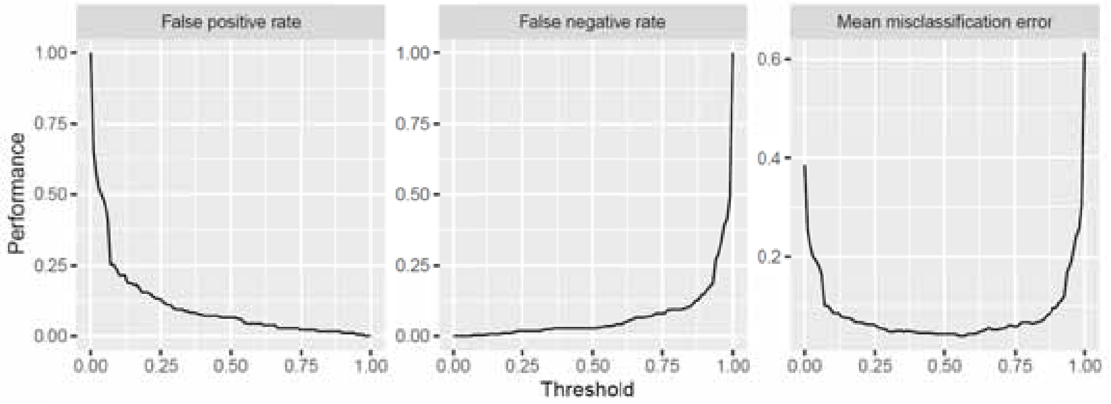

Figure S10. Performances of false positive rate, false negative rate, and mean misclassification error with threshold.

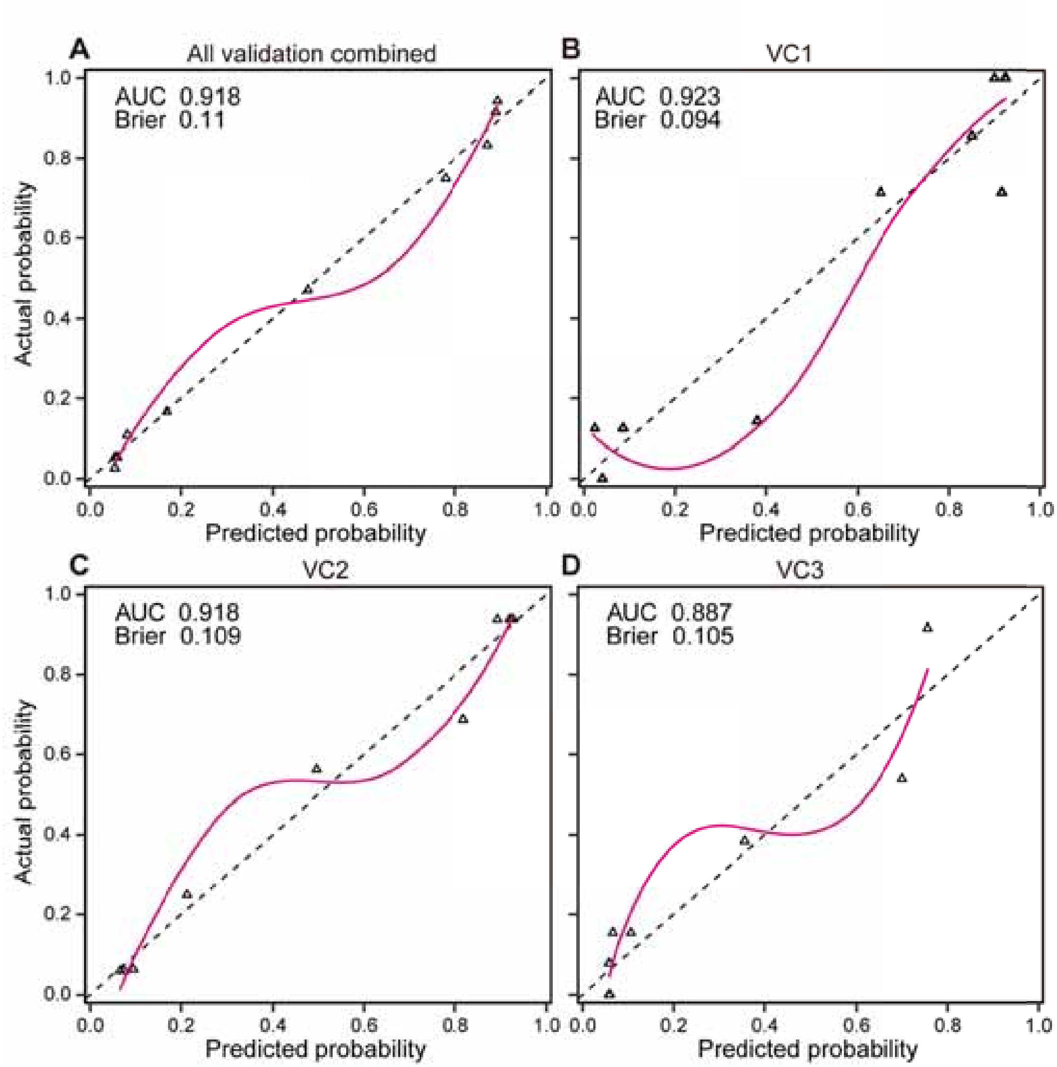

Figure S11. Calibration curves of TEPOC in validation cohorts. Calibration curves of TEPOC in combined validation cohort (**A**), validation cohort 1 (**B**), validation cohort 2 (**C**), and validation cohort 3 (**D**), respectively. The triangle represents the observation group. The dashed line is the ideal calibration curve. Red curve is the fitted linear logistic calibration curve generated using a loess smooth. AUC, Area under the curve. Brier, Brier score.

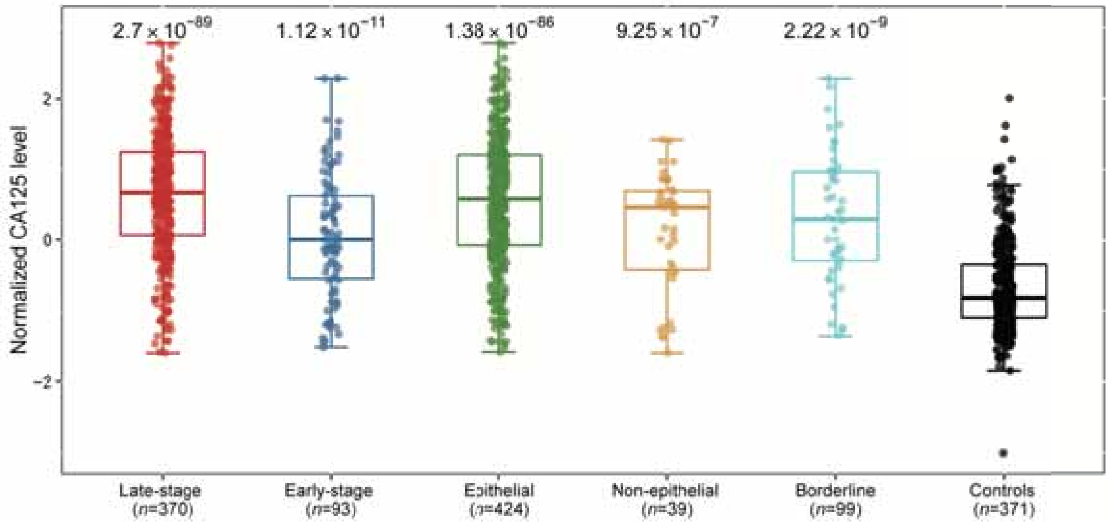

Figure S12. CA125 levels in ovarian cancer subgroups and controls. Boxplots represent median value, with lower and upper hinges corresponding to the 25th and 75th percentiles, and lower and upper whiskers extending from the hinge to the smallest and largest value at most 1.5× interquartile range of the hinge, respectively. The normalized CA125 levels in ovarian cancer subgroups were compared with those of controls. Two-sided Student's *t*-test. CA125, cancer antigen 125. OC, ovarian cancer. Controls included patients with benign adnexal masses and healthy women.

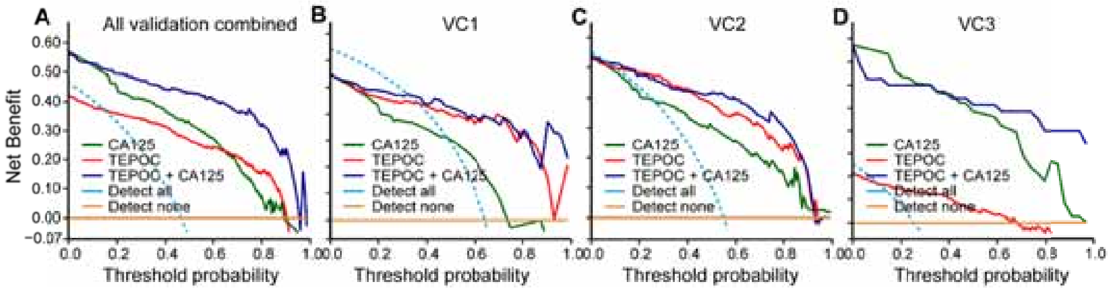

Figure S13. Decision curve analysis across validation cohorts. The decision curves show the clinical usefulness of the models in detecting ovarian cancer in the validation cohorts. The blue line represents the assumption that all patients have ovarian cancer (i.e. detect all), while the yellow line represents the assumption that no patients have ovarian cancer (i.e. detect none). The other colored lines depict the net benefit of using models to detect ovarian cancer.

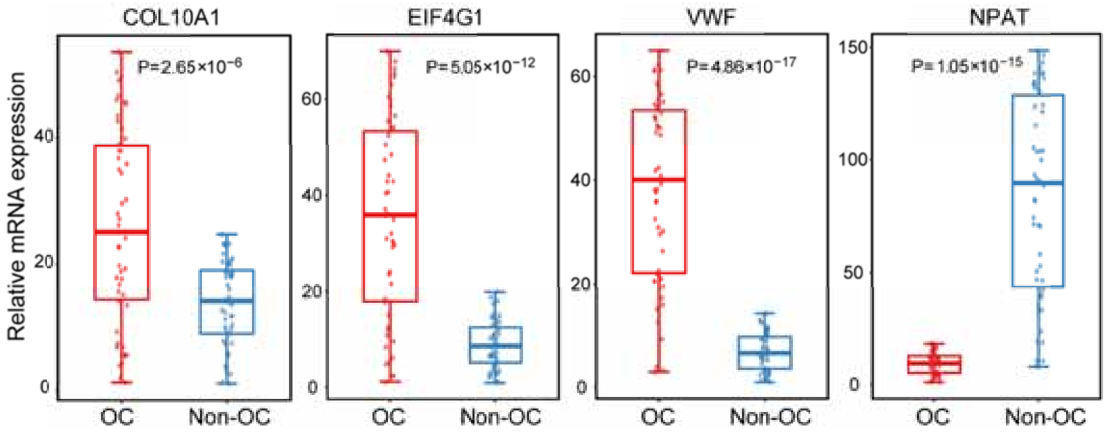

Figure S14. Representative differentially expressed genes between OC and non-OC samples. Box and jitter plots showing four representative differentially expressed genes between OC (n = 50) and non-OC (n = 50) samples. The center line represents the median of relative expression. Box limits represent upper and lower quartiles. Whiskers represent 1.5 times interquartile range. Wilcoxon test was used in the univariate comparison between groups and a two-tailed $p < 0.05$ was considered as statistically significant. OC, ovarian cancer; Non-OC, non-ovarian cancer.

References

1. Best, M., et al., Swarm Intelligence-Enhanced Detection of Non-Small-Cell Lung Cancer Using Tumor-Educated Platelets. Cancer cell, 2017. 32(2): p. 238-252.e9.
2. Best, M., et al., RNA sequencing and swarm intelligence-enhanced classification algorithm development for blood-based disease diagnostics using spliced blood platelet RNA. Nature protocols, 2019. 14(4): p. 1206-1234.
3. Love, M., W. Huber, and S. Anders, Moderated estimation of fold change and dispersion for RNA-seq data with DESeq2. Genome biology, 2014. 15(12): p. 550.
4. Leek, J., svaseq: removing batch effects and other unwanted noise from sequencing data. Nucleic acids research, 2014. 42(21).
5. Ritchie, M., et al., limma powers differential expression analyses for RNA-sequencing and microarray studies. Nucleic acids research, 2015. 43(7): p. e47.
6. Byron, S.A., et al., Translating RNA sequencing into clinical diagnostics: opportunities and challenges. Nat Rev Genet, 2016. 17(5): p. 257-71.
7. Li, W., J. Feng, and T. Jiang, IsoLasso: a LASSO regression approach to RNA-Seq based transcriptome assembly. J Comput Biol, 2011. 18(11): p. 1693-707.
8. Mundra, P.A. and J.C. Rajapakse, SVM-RFE with MRMR filter for gene selection. IEEE Trans Nanobioscience, 2010. 9(1): p. 31-7.
9. Huang, Y., et al., To permute or not to permute. Bioinformatics, 2006. 22(18): p. 2244-8.
10. Best, M., et al., RNA-Seq of Tumor-Educated Platelets Enables Blood-Based Pan-Cancer, Multiclass, and Molecular Pathway Cancer Diagnostics. Cancer cell, 2015. 28(5): p. 666-676.

Supplementary Tables

Supplementary Table S1. Compositions of benign adnexal masses.

Histology	Training cohort	Validation cohorts
Serous cystadenoma, n (%)	16 (8.8)	9 (8.4)
Mucinous cystadenoma, n (%)	2 (1.1)	1 (0.9)
Cyst adenofibroma, n (%)	17 (9.3)	10 (9.3)
Fibroma, n (%)	10 (5.5)	6 (5.6)
Steroid cell tumor, n (%)	2 (1.1)	1 (0.9)
Struma ovarii, n (%)	2 (1.1)	1 (0.9)
Tuberculous granuloma, n (%)	1 (0.6)	0 (0)
Mature cystic teratoma, n (%)	20 (11.0)	12 (11.2)
Ovarian endometriotic cysts, n (%)	57 (31.3)	37 (34.6)
Hydrosalpinx, n (%)	8 (4.4)	5 (4.7)
Ovarian corpus luteum cyst, n (%)	7 (3.8)	2 (1.9)
Paraovarian cyst, n (%)	2 (1.1)	0 (0)
Other cysts, n (%)	38 (20.9)	23 (21.5)
Total, n (%)	182 (100)	107 (100)

Supplementary Table S2. Gene list and description of TEPOC.

	Ensemble Gene Id	Hgnc Symbol		Ensemble Gene Id	Hgnc Symbol		Ensemble Gene Id	Hgnc Symbol
1	ENSG00000003436	TFPI	35	ENSG00000123500	COL10A1	69	ENSG00000167985	SDHAF2
2	ENSG00000005249	PRKAR2B	36	ENSG00000125354	SEPTIN6	70	ENSG00000168385	SEPTIN2
3	ENSG00000008018	PSMB1	37	ENSG00000125503	PPP1R12C	71	ENSG00000169567	HINT1
4	ENSG00000037042	TUBG2	38	ENSG00000125534	PPDPF	72	ENSG00000169762	TAPT1
5	ENSG00000065534	MYLK	39	ENSG00000127540	UQCR11	73	ENSG00000171314	PGAM1
6	ENSG00000067167	TRAM1	40	ENSG00000128311	TST	74	ENSG00000175063	UBE2C
7	ENSG00000071127	WDR1	41	ENSG00000130475	FCHO1	75	ENSG00000175387	SMAD2
8	ENSG00000080371	RAB21	42	ENSG00000131389	SLC6A6	76	ENSG00000177169	ULK1
9	ENSG00000087053	MTMR2	43	ENSG00000131966	ACTR10	77	ENSG00000177565	TBL1XR1
10	ENSG00000087470	DNM1L	44	ENSG00000132300	PTCD3	78	ENSG00000177697	CD151
11	ENSG00000089009	RPL6	45	ENSG00000132475	H3-3B	79	ENSG00000177868	SVBP
12	ENSG00000091592	NLRP1	46	ENSG00000132718	SYT11	80	ENSG00000178562	CD28
13	ENSG00000100266	PACSIN2	47	ENSG00000136205	TNS3	81	ENSG00000181690	PLAG1
14	ENSG00000100614	PPM1A	48	ENSG00000138758	SEPTIN11	82	ENSG00000184226	PCDH9
15	ENSG00000100644	HIF1A	49	ENSG00000140450	ARRDC4	83	ENSG00000184602	SNN
16	ENSG00000100722	ZC3H14	50	ENSG00000140455	USP3	84	ENSG00000184640	SEPTIN9
17	ENSG00000102158	MAGT1	51	ENSG00000142168	SOD1	85	ENSG00000184838	PRR16
18	ENSG00000105499	PLA2G4C	52	ENSG00000143033	MTF2	86	ENSG00000185305	ARL15
19	ENSG00000108100	CCNY	53	ENSG00000145335	SNCA	87	ENSG00000197601	FAR1
20	ENSG00000110090	CPT1A	54	ENSG00000146731	CCT6A	88	ENSG00000198626	RYR2
21	ENSG00000110324	IL10RA	55	ENSG00000148481	MINDY3	89	ENSG00000212907	MT-ND4L
22	ENSG00000110799	VWF	56	ENSG00000149308	NPAT	90	ENSG00000226950	DANCR
23	ENSG00000110848	CD69	57	ENSG00000151789	ZNF385D	91	ENSG00000233822	H2BC15
24	ENSG00000111328	CDK2AP1	58	ENSG00000151838	CCDC175	92	ENSG00000233954	UQCRHL
25	ENSG00000112651	MRPL2	59	ENSG00000152926	ZNF117	93	ENSG00000234231	ANAPC1P4
26	ENSG00000114127	XRN1	60	ENSG00000163220	S100A9	94	ENSG00000236304	*
27	ENSG00000114867	EIF4G1	61	ENSG00000163320	CGGBP1	95	ENSG00000240497	*
28	ENSG00000116717	GADD45A	62	ENSG00000163812	ZDHHC3	96	ENSG00000249936	RAC1P2
29	ENSG00000117054	ACADM	63	ENSG00000165698	SPACA9	97	ENSG00000251562	MALAT1
30	ENSG00000118276	B4GALT6	64	ENSG00000166165	CKB	98	ENSG00000253819	LINC01151
31	ENSG00000118418	HMGN3	65	ENSG00000166887	VPS39	99	ENSG00000253982	*
32	ENSG00000119801	YPEL5	66	ENSG00000167005	NUDT21	100	ENSG00000254893	RAP1BL
33	ENSG00000122008	POLK	67	ENSG00000167740	CYB5D2	101	ENSG00000255364	SMILR
34	ENSG00000122643	NT5C3A	68	ENSG00000167912	*	102	ENSG00000257365	FNTB

* Novel transcripts.

For gene descriptions, please see the attached Excel file named Expanded Supplementary Table S2.

Supplementary Table S3. Performance of TEPOC and CA125 to detect ovarian cancer in HGSOC cohort.

	**AUC (95% CI)**	**ACC (95% CI), %**	**SN (95% CI), %**	**SP (95% CI), %**	**PPV (95% CI), %**	**NPV (95% CI), %**	**Kappa**	**F1**	**AUC *P* value**
TEPOC	0.903 (0.856–0.951)	83.1 (76.9–88.1)	91.5 (83.2–96.5)	76.6 (67.5–84.3)	75.0 (65.3–83.1)	92.1 (84.5–96.8)	0.664	0.824	$P = 0.11$
CA125	0.839 (0.776–0.902)	78.3 (71.6–84.1)	85.0 (75.3–92.0)	73.0 (63.2–81.4)	71.6 (61.4–80.4)	85.9 (76.6–92.5)	0.569	0.777	-
TEPOC+CA125	0.934 (0.893–0.974)	88.9 (83.4–93.1)	92.5 (84.4–97.2)	86.0 (77.6–92.1)	84.1 (74.8–91.0)	93.5 (86.3–97.6)	0.777	0.881	$P = 0.009$

Predictions of TEPOC and the combination were compared with those of CA125 using a two-sided DeLong's test. *Abbreviations*: TEPOC, tumor-educated platelet-derived gene panel of ovarian cancer. CA125, cancer antigen 125. HGSOC, high grade serous ovarian cancer. TEPOC+CA125, a combinatory diagnosis of TEPOC and CA125. AUC, area under the curve. ACC, accuracy. SN, sensitivity. SP, specificity. PPV, positive predictive value. NPV, negative predictive value. CI, confidence interval.

Supplementary Table S4. Performance of TEPOC and CA125 to detect ovarian cancer with pre-specified specificity at 90% with all non-OC as controls.

	AUC (95% CI)	ACC (95% CI), %	SN (95% CI), %	SP (95% CI), %	PPV (95% CI), %	NPV (95% CI), %	Kappa	F1
**Early-stage**								
TEPOC	0.893 (0.842–0.944)	86.7 (81.9–90.7)	70.7 (54.5–83.9)	90.0 (85.0–93.6)	58.0 (43.2–71.8)	94.0 (89.7–96.8)	0.449	0.561
CA125	0.745 (0.657–0.832)	75.2 (67.6–81.7)	31.7 (18.1–48.1)	90.0 (83.7–95.2)	54.2 (32.8–74.4)	78.9 (71.0–85.5)	0.335	0.540
TEPOC+CA125	0.883 (0.820–0.946)	85.4 (78.8–90.5)	73.2 (57.1–85.8)	90.0 (82.6–94.5)	71.4 (55.4–84.3)	90.4 (83.5–95.1)	0.559	0.682
**Borderline**								
TEPOC	0.946 (0.917–0.975)	89.7 (85.1–93.2)	88.2 (72.5–96.7)	90.0 (85.0–93.6)	58.8 (44.2–72.4)	97.9 (94.7–99.4)	0.542	0.627
CA125	0.773 (0.682–0.863)	78.0 (70.5–84.3)	35.3 (19.7–53.5)	90.0 (83.7–95.2)	52.2 (30.6–73.2)	82.7 (75.0–88.8)	0.371	0.549
TEPOC+CA125	0.953 (0.922–0.984)	90.0 (84.0–94.3)	91.2 (76.3–98.1)	90.0 (82.6–94.5)	72.1 (56.3–84.7)	97.2 (92.0–99.4)	0.668	0.756
**Non-epithelial**								
TEPOC	0.921 (0.873–0.970)	87.8 (80.9–92.9)	78.3 (56.3–92.5)	90.0 (82.5–94.8)	62.1 (42.3–79.3)	95.1 (88.9–98.4)	0.463	0.585
CA125	0.741 (0.643–0.838)	78.6 (70.6–85.3)	26.1 (10.2–48.4)	90.0 (82.5–94.8)	35.3 (14.2–61.7)	85.1 (77.2–91.1)	0.243	0.418
TEPOC+CA125	0.929 (0.881–0.976)	88.5 (81.8–93.4)	82.6 (61.2–95.0)	90.0 (82.5–94.8)	63.3 (43.9–80.1)	96.0 (90.2–98.9)	0.575	0.667
**High grade**								
TEPOC	0.927 (0.892–0.962)	88.1 (83.9–91.6)	83.9 (74.5–90.9)	90.0 (85.0–93.6)	77.7 (67.9–85.6)	93.0 (88.6–96.1)	0.672	0.782
CA125	0.842 (0.783–0.900)	75.6 (69.1–81.4)	55.3 (44.1–66.1)	90.0 (83.7–95.2)	81.0 (68.6–90.1)	73.4 (65.4–80.5)	0.576	0.775
TEPOC+CA125	0.942 (0.907–0.978)	89.1 (83.9–93.0)	88.2 (79.4–94.2)	90.0 (82.6–94.5)	86.2 (77.1–92.7)	91.2 (84.5–95.7)	0.770	0.873

Abbreviations: TEPOC, tumor-educated platelet-derived gene panel of ovarian cancer. CA125, cancer antigen 125. TEPOC+CA125, a combinatory diagnosis of TEPOC and CA125. Non-OC, non-ovarian cancer. AUC, area under the curve. ACC, accuracy. SN, sensitivity. SP, specificity. PPV, positive predictive value. NPV, negative predictive value. CI, confidence interval.

Supplementary Table S5. Performance of TEPOC and the combination model to detect ovarian cancer in validation cohorts.

	AUC (95% CI)	ACC (95% CI), %	SN (95% CI), %	SP (95% CI), %	PPV (95% CI), %	NPV (95% CI), %	AUC *P* value
**All validation**							
TEPOC	0.918 (0.889-0.948)	83.8 (79.6-87.4)	85.3 (78.7-90.4)	82.7 (76.9-87.6)	78.7 (71.7-84.6)	88.2 (82.8-92.4)	-
TEPOC+CA125	0.922 (0.889-0.955)	85.9 (81.2-89.8)	86.4 (79.9-91.4)	85.3 (77.6-91.2)	88.7 (82.5-93.3)	82.5 (74.5-88.8)	$P = 0.870$
**VC1**							
TEPOC	0.923 (0.855-0.990)	84.9 (74.6-92.2)	95.0 (83.1-99.4)	72.7 (54.5-86.7)	80.9 (66.7-90.9)	92.3 (74.9-99.1)	-
TEPOC+CA125	0.955 (0.912-0.997)	87.7 (77.9-94.2)	92.5 (79.6-98.4)	81.8 (64.5-93.0)	86.0 (72.1-94.7)	90.0 (73.5-97.9)	$P = 0.058$
**VC2**							
TEPOC	0.918 (0.872-0.963)	84.0 (77.4-89.2)	86.2 (77.1-92.7)	81.3 (70.7-89.4)	84.3 (75.0-91.1)	83.6 (73.0-91.2)	-
TEPOC+CA125	0.939 (0.901-0.977)	87.7 (81.6-92.3)	89.7 (81.3-95.2)	85.3 (75.3-92.4)	87.6 (79.0-93.7)	87.7 (77.9-94.2)	$P = 0.011$
**VC3**							
TEPOC	0.887 (0.813-0.960)	82.9 (75.3-89.0)	69.0 (49.2-84.7)	87.0 (78.8-92.9)	60.6 (42.1-77.1)	90.6 (82.9-95.6)	-
TEPOC+CA125	0.917 (0.824-1.000)	74.3 (56.7-87.5)	66.7 (46.0-83.5)	100.0 (63.1-100.0)	100.0 (81.5-100.0)	47.1 (23.0-72.2)	$P = 0.623$

Predictions of TEPOC were compared with those of the combination model using a two-sided DeLong's test. Abbreviations: TEPOC, tumor-educated platelet-derived gene panel of ovarian cancer. TEPOC+CA125, the combination of TEPOC and CA125. AUC, area under the curve. ACC, accuracy. SN, sensitivity. SP, specificity. PPV, positive predictive value. NPV, negative predictive value. CI, confidence interval.

Supplementary Table S6. List of the primer sequences.

Gene	Forward sequence	Reverse sequence
ACTB	TTAGTTGCGTTACACCCTTTC	GCTGTCACCTTCACCGTTC
COL10A1	GATACCAAATGCCCACAGG	CCTCTTACTGCTATACCTTTACTC
EIF4G1	AAACCCAGGACCTATTCCG	CTTGCTTCATCAGCTGCTG
VWF	CACTGAAGCGTGATGAGAC	CCCAGAAGTACTCTCCTCTC
NPAT	ACTTTCTCAGATCAGGAGCA	TCTGCAATTCCAGTTCTCG
